# Supplementary material for: Phospho-signaling couples polar asymmetry and proteolysis within a membraneless microdomain in Caulobacter crescentus
Source: Nat Commun. 2024 Oct 28;15:9282. doi: 10.1038/s41467-024-53395-y (PMC11519897; doi:10.1038/s41467-024-53395-y)
Supplement: Supplementary file 1 — Supplementary Information [file 41467_2024_53395_MOESM1_ESM.pdf]

Phospho-signaling couples polar asymmetry and proteolysis within a membraneless microdomain in *Caulobacter crescentus*.

Yasin M Ahmed[1], Logan M Brown[2], Krisztina Varga[2], Grant R Bowman[1*]

1. Department of Molecular Biology, University of Wyoming, Laramie, WY 82071, USA
2. Department of Molecular, Cellular, and Biomedical Sciences, University of New Hampshire, Durham, NH 03824, USA

Contents:

1. Supplementary Figures
2. Supplementary Tables
3. Supplementary References

Supplementary Figures

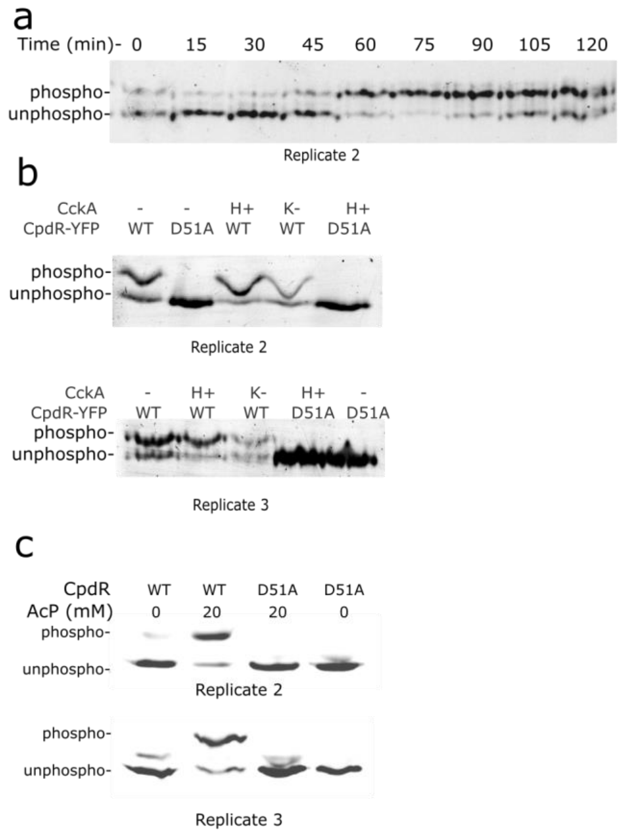

Supplementary Fig. 1| Phos-tag gel replicates for *C. crescentus* and *in vitro* experiments.

a, Additional replicate for Fig. 1c. **b,** Additional replicates for Fig. 1f. **c,** Additional replicates for Fig. 3c. Source data are provided as a Source Data file.

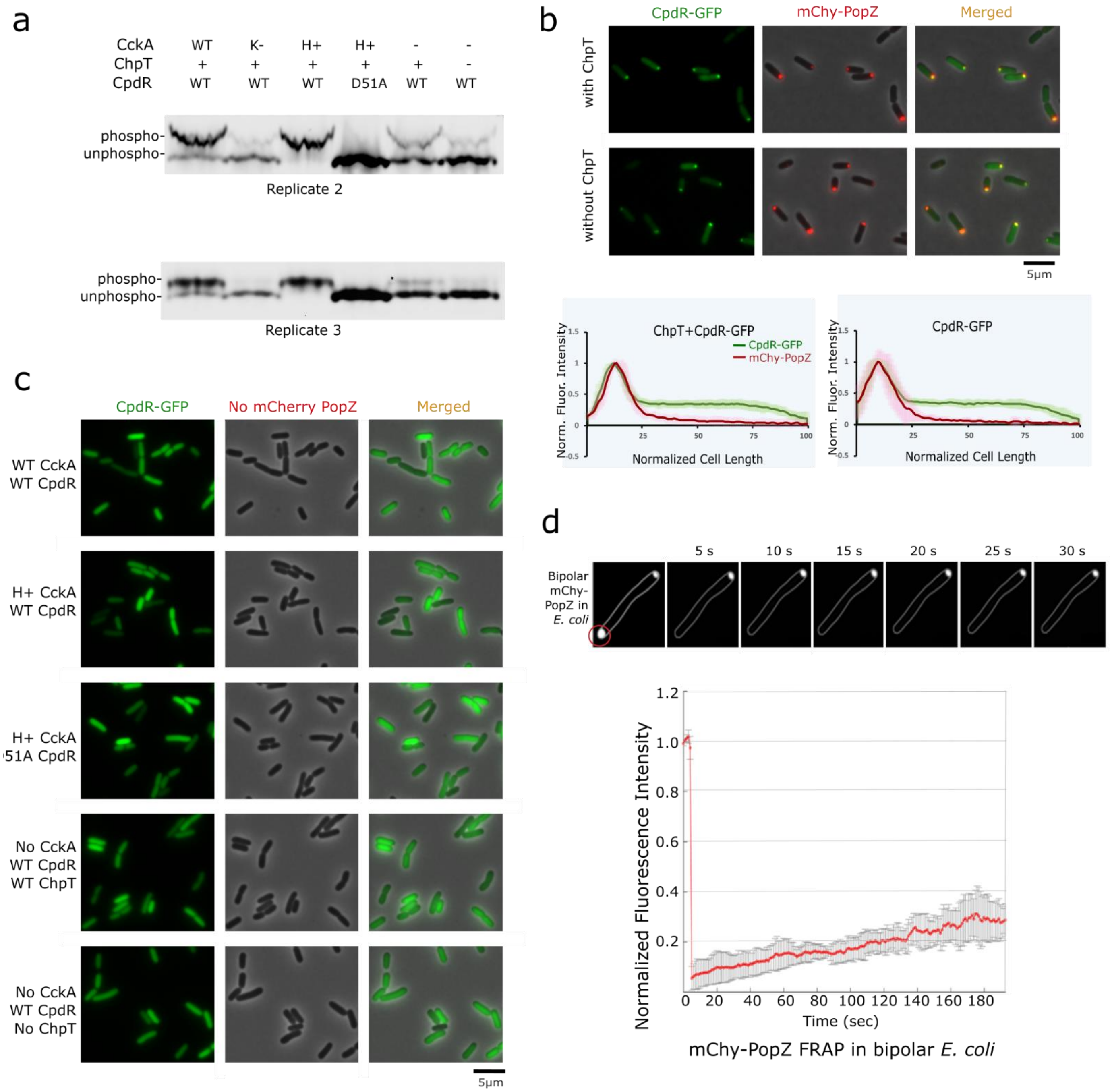

Supplementary Fig. 2| Protein localization and dynamics in *E. coli*.

a, Additional replicates for Fig. 2b. **b,** *E. coli* co-expression assay, with mChy-PopZ and CpdR-GFP expressed in the presence or absence of the intermediary phosphotransferase ChpT. mChy-PopZ and CpdR-GFP were observed by microscopy and normalized fluorescence intensities were plotted against cell length (graphs). **c,** *E. coli* co-expression assay, with different CckA, ChpT and

CpdR-GFP variants expressed in absence of mChy-PopZ. Images were taken in mChy and GFP channel. **d,** FRAP analysis of mChy-PopZ in *E. coli* cells with bipolar foci. Recovery of fluorescence was plotted against time in seconds. Source data are provided as a Source Data file.

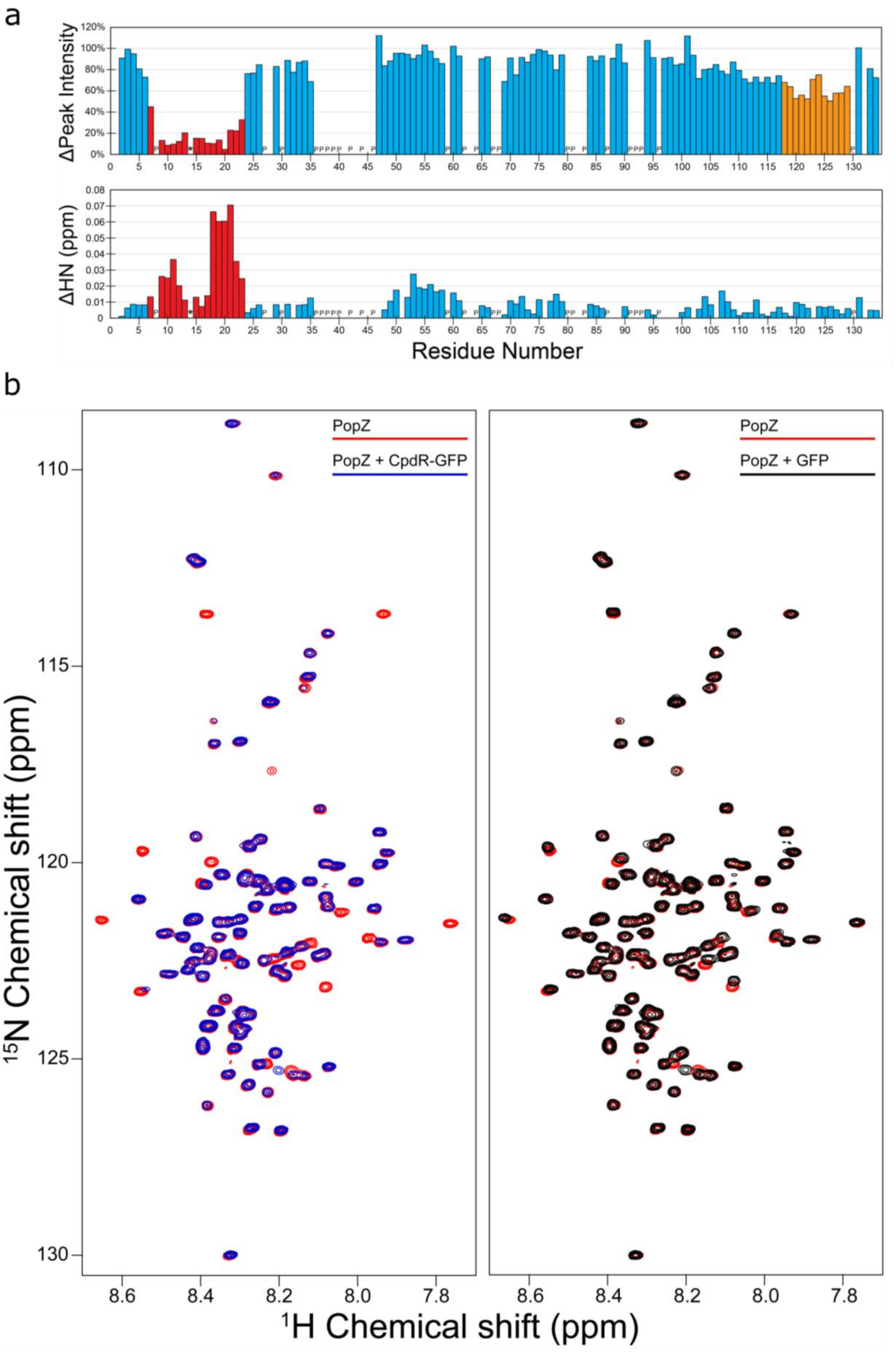

a
ΔPeak Intensity
ΔHN (ppm)
Residue Number
b
PopZ
PopZ + CpdR-GFP
PopZ
PopZ + GFP
15N Chemical shift (ppm)
1H Chemical shift (ppm)

Supplementary Fig. 3|Chemical shift perturbations of PopZΔ$^{134–177}$ upon binding to CpdR-GFP.

a, Top graph: Signal attenuation resulting from the addition of 750 µM CpdR-GFP to 50 µM PopZΔ$^{134–177}$. The changes in peak intensities were calculated as percentages relative to the peak intensities at the 0 µM CpdR-GFP titration point (i.e., PopZΔ$^{134–177}$ peak intensity in the absence of binding protein). Bottom graph: ΔHN combined chemical shift perturbations of PopZΔ$^{134–177}$ (50 µM) upon binding to 750 µM CpdR-GFP as calculated by Equation 1. The most pronounced shifts, indicated in red, align well with the previously determined binding region of PopZΔ$^{134–177}$. A potential secondary weak binding region is highlighted in orange in the top spectrum, where the peak intensity attenuation slightly exceeded one standard deviation, however no significant ΔHN was observed for these residues. Note that Pro residues cannot be observed in ^{1}H-^{15}N HSQC spectra and are indicated with a "P" on the x-axis in both graphs. A few other resonances were either overlapped (indicated with an asterisk), could not be assigned, or were missing under the buffer conditions. **b,** Left panel: ^{1}H-^{15}N TROSY-HSQC NMR spectra overlay of 50 µM ^{15}N-enriched PopZΔ$^{134–177}$ with and without its binding partner, CpdR-GFP: PopZΔ$^{134–177}$ only (i.e., 0 µM CpdR-GFP) (red spectrum) and PopZΔ$^{134–177}$ with 750 µM CpdR-GFP (blue spectrum). Right panel: In a control experiment, 750 µM GFP was added to PopZΔ$^{134–177}$ (black spectrum) and overlayed with PopZΔ$^{134–177}$ spectrum (i.e., no binding partner, red spectrum). While PopZΔ$^{134–177}$ exhibits large chemical shift perturbations for selected residues upon the addition of CpdR-GFP, only minor changes were observed in the spectrum upon the addition of the same concentration of GFP, indicating that CpdR is the primary binding partner of PopZΔ$^{134–177}$.

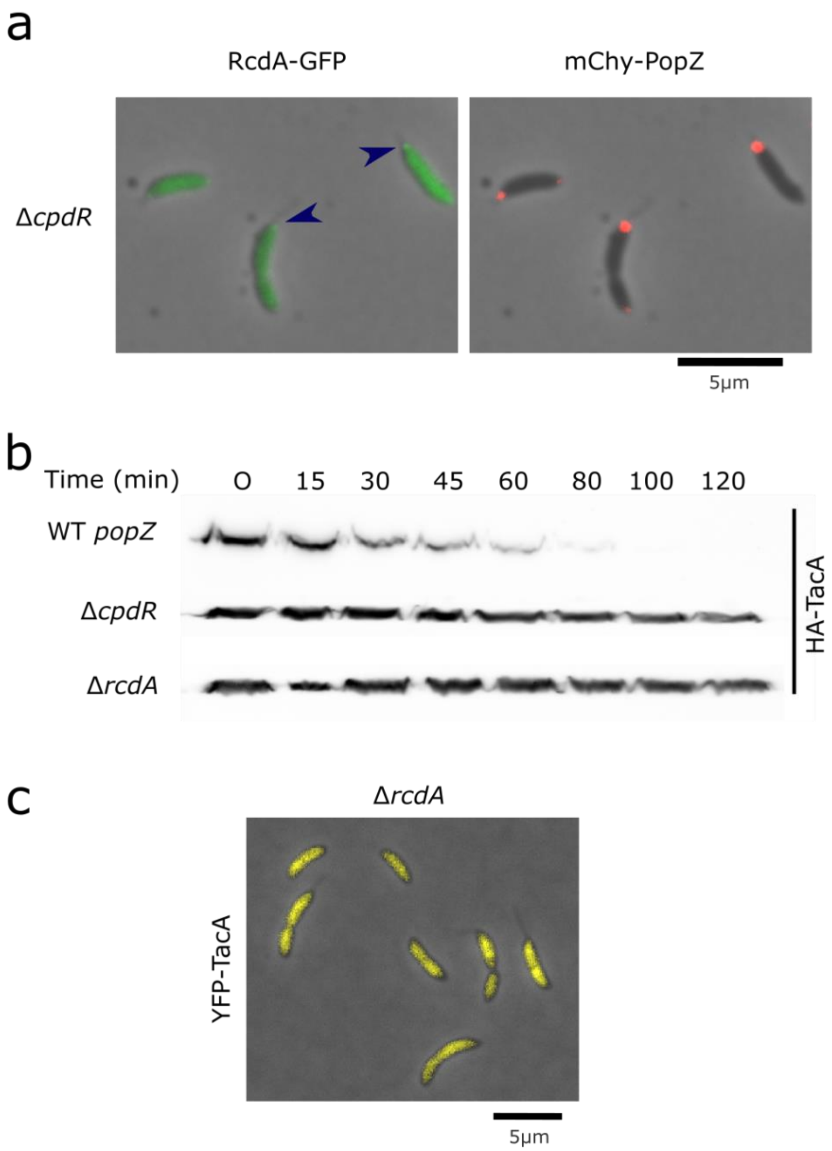

Supplementary Fig. 4| TacA requires CpdR and RcdA adaptors for proteolysis and polar localization.

a, Faint foci of RcdA-GFP in Δ*cpdR* strain (arrowheads) are difficult to discern and quantify. **b,** Degradation of HA-TacA following inducer wash-out in Δ*popZ*, Δ*cpdR*, and Δ*rcdA C. crescentus* strain backgrounds, observed by western blotting with α-HA antibody. **c,** YFP-TacA localization in Δ*rcdA C. crescentus* strain background. Source data are provided as a Source Data file.

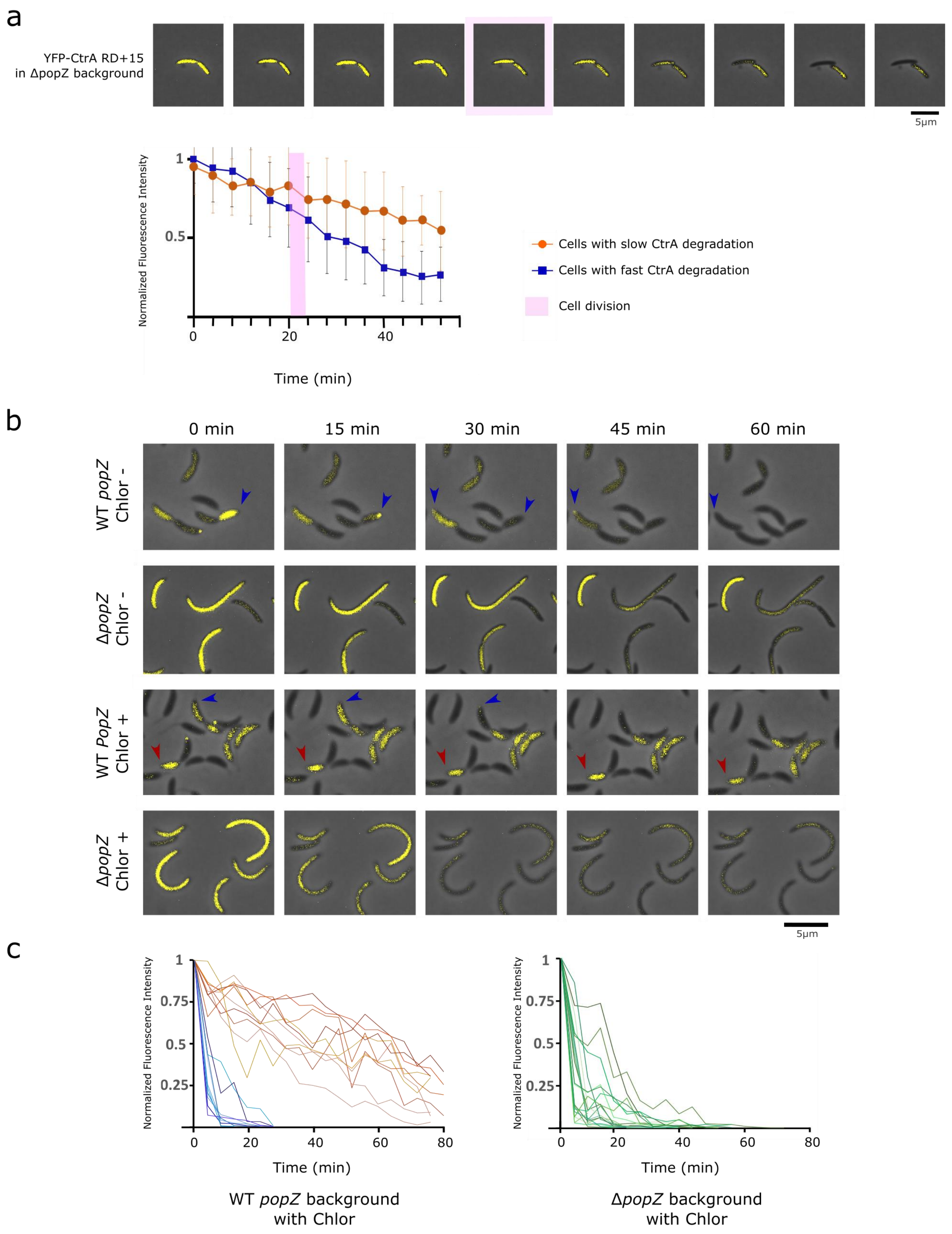
a
YFP-CtrA RD+15
in ΔpopZ background
5μm
Normalized Fluorescence Intensity
1
0.5
0
20
40
Time (min)
Cells with slow CtrA degradation
Cells with fast CtrA degradation
Cell division
b
0 min
15 min
30 min
45 min
60 min
WT popZ Chlor -
ΔpopZ Chlor -
WT PopZ Chlor +
ΔpopZ Chlor +
5μm
c
Normalized Fluorescence Intensity
1
0.75
0.5
0.25
0
20
40
60
80
Time (min)
WT popZ background with Chlor
ΔpopZ background with Chlor

Supplementary Fig. 5| Substrate degradation in individual cells, in the presence and absence of chloramphenicol.

a, Time-lapse images of YFP-tagged substrate localization in a Δ*popZ C. crescentus* background, at 4 minute intervals. Pink bar idicates the time of cell separation. After accounting for photobleaching and temporally aligning the cells with respect to the time of cell separation (n=20), average fluorescence intensities for the faster and slower YFP-CtrA RD+15 degrading cells, normalized to maximum fluorescence intensity, were plotted against time (line graphs, bar = stadard deviation). **b,** YFP-CtrA RD+15 distribution in WT and Δ*popZ C. crescentus* stain backgrounds with and without chloramphenicol (chlor) treatment. Blue arrowheads mark cells that exhibit YFP-CtrA RD+15 polar foci and degradation. Red arrows mark cells that do not exhibit YFP-CtrA RD+15 polar foci or degradation. **c,** Average YFP fluorescence intensities of different cell bodies at 4 minute intervals, normalized to maximum fluorescence intensity, were plotted against time (line graphs), after accounting for photobleaching. Blue lines trace fluorescence intinsity in WT cells that exhibit polar YFP foci during degradation (n=10), orange lines trace fluorescence intensity in WT cells that do not exhibit polar foci (n=10), and green lines trace fluorescence intensity in Δ*popZ* cells (n=20). Source data are provided as a Source Data file.

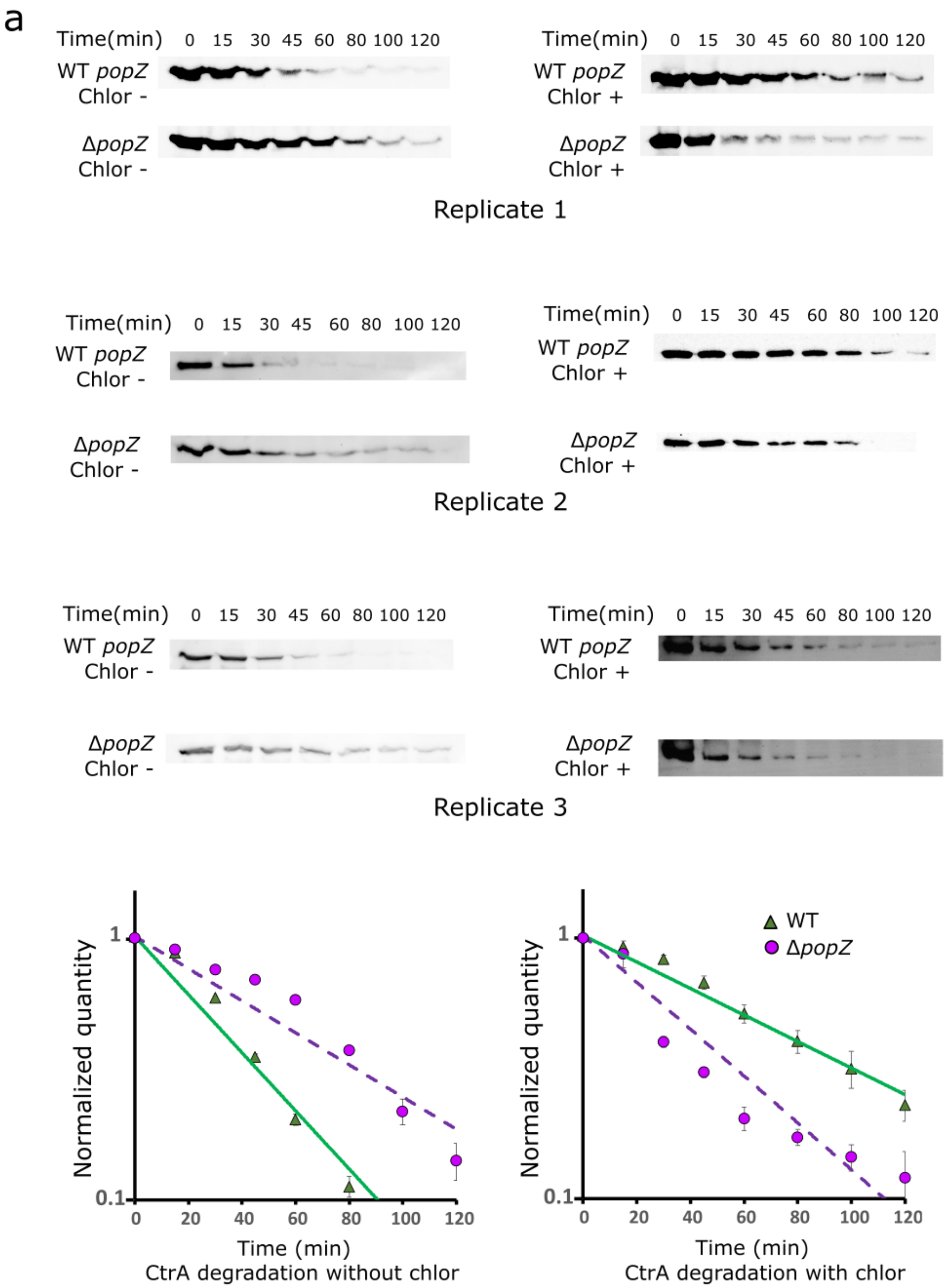

Supplementary Fig. 6| Substrate degradation at the population level, in the presence and absence of chloramphenicol.

a, Degradation of YFP-CtrA RD+15 following inducer wash-out in wildtype and Δ*popZ* strain backgrounds in the presence or absence of chloamphenicol treatment. Western blotting was performed with α-GFP antibody (GeneTex GTX113617) at 1:3000. Three relicates are presented, and the average band intensities are plotted against time (bar=standard deviation). Source data are provided as a Source Data file.

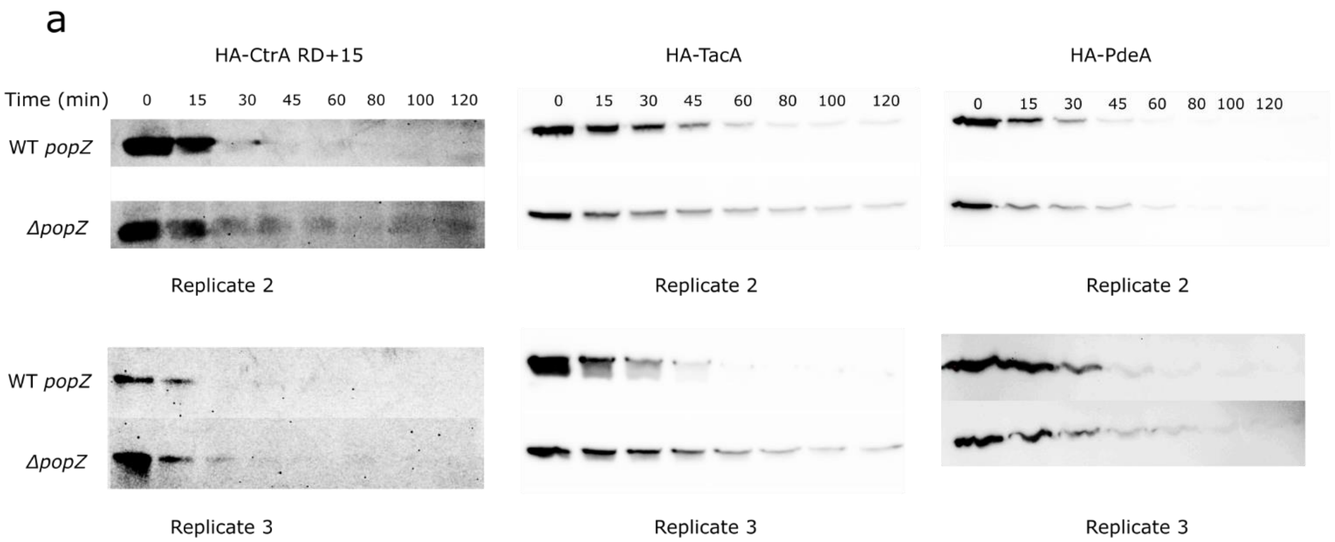

Supplementary Fig. 7| Degradation of HA-tagged proteolysis substrates following inducer wash-out.

a, Additional replicates for Fig. 5c. Source data are provided as a Source Data file. Source data are provided as a Source Data file.

a

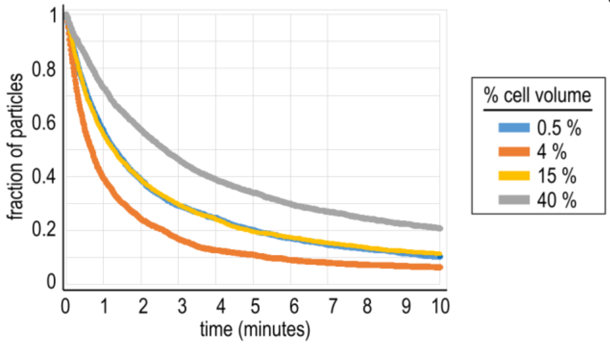

b

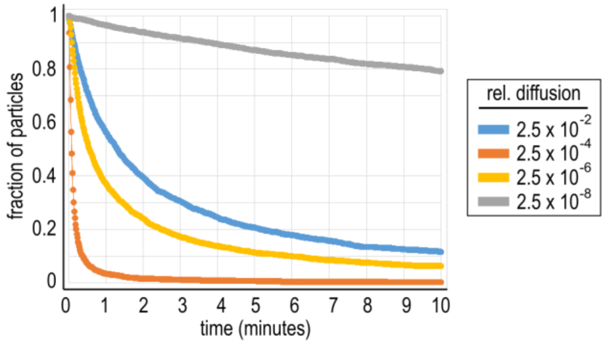

c

Cell Type	% at cell pole			Time to 75% proteolysis (min)	
	ClpXP	CpdR	RcdA	Sub1	Sub2
normal	6.4	14.5	14.3	1.4	3.6
Δ*popZ*	0.5	0.5	0.5	1.6	7.4
$CpdR^{mut}$	0.6	0.6	14.3	1.6	7.4
Δ*rcdA*	6.2	14.4	--	1.3	∞
Δ*cpdR*	0.5	--	14.4	∞	∞
$RcdA^{mut}$	6.5	14.5	0.6	1.3	5.9
1/160 difc	25.4	39.8	39.9	1.3	2.9
2% vol	24.7	42.1	42.0	1.3	3.0

Supplementary Fig. 8| Conceptual models of substrate proteolysis in polar microdomains.

a-b, Three-dimensional reaction-diffusion simulations, as shown in Fig. 5a-b, except using different parameters. **a**, Cells with different sizes of polar microdomains, expressed as a percentage of total cell volume. 0.5% approximates the physiological size of PopZ microdomains *in C. crescentus*. At extremely large microdomain volumes, the reaction slows because particles are less concentrated in a large compartment with a relatively slow particle diffusion rate. **b**, Cells with polar microdomains that have different particle diffusion rates. 2.5 x 10^{-2} (or 1/40) approximates the physiological difference in protein diffusion rates in PopZ microdomains versus bulk cytoplasm in *C. crescentus*. Up to a point, slower diffusion rates lead to higher particle concentration and faster reaction rates. However, when particle diffusion in polar microdomains is extremely slow, most particles become stuck just below the microdomain surface and their collision frequency is low. **c**, Performance of the *C. crescentus* substrate proteolysis model, presented in Fig. 5c, under different conditions. The chart columns show the polar concentration of ClpXP, CpdR, and RcdA, expressed as the average percentage of total particles in polar microdomains over the course of the simulation, and the time needed the degrade 50% of substrates Sub1 and Sub2. "Normal" reports on a simulation that includes all components, with microdomain size, diffusion rates, and binding affinities at baseline values that approximate physiological conditions. Δ*popZ*, Δ*cpdR*, Δ*rcdA*, report on

simulations run in the absence of these components. $CpdR^{mut}$ and $RcdA^{mut}$ report on the effects of selectively removing the interaction between the indicated adaptor protein and PopZ, by assigning them the rapid cytoplasmic rate of diffusion when that are located in polar microdomains. $1/160^{th}$ difc and 2% vol report on simulations with slower polar microdomain diffusion rates or larger polar microdomain sizes, as described in Fig. 5b and 5a, respectively. Source data are provided as a Source Data file.

Supplementary Tables

Supplementary Table 1

Strain name	Genotype / Background	Plasmid description		Source
		Replicating	Integrating	
**GB#1194**	*Caulobacter* wildtype synchronizable strain NA1000 CB15N			[1]
**GB#1081**	*Caulobacter popZ*::*specR*			[2]
**GB#1007**	*Caulobacter popZ::mChy-popZ*			[3]
**GB#264**	*Caulobacter* WT; GB#1194	pMR10 YFP-CtrA RD+15		[4]
**GB#228**	*Caulobacter* WT; GB#1194	pMR11 pCpdR-CpdR-YFP		[5]
**GB#944**	*E. coli* BL21 DE3	pBad-mChy PopZ		[6]
**GB#169**	*E. coli* Rosetta	pET28a-6XHis-PopZ		[2]
**JH#66**	*E. coli* DH5α	pBAD-6XHis-GFP		[7]
**GB#1969**		pACYC-CpdR-TEV-msfGFP-6XHis		this study
**GB#1970**		pACYC-CpdR$_{D51A}$-TEV-msfGFP-6XHis		this study
**GB#2022**	*E. coli* MG1655 DE3 *clpXP*::*kanR*			this study
**GB#1971**	*E. coli* MG1655 DE3 *clpXP*::*kanR*	pACYC-CpdRGFP-ChpT-CckA WT + pBAD-mCherry PopZ		this study
**GB#1972**	*E. coli* MG1655 DE3 *clpXP*::*kanR*	pACYC-CpdRGFP-ChpT-CckA H+ + pBAD-mCherry PopZ		this study
**GB#1973**	*E. coli* MG1655 DE3 *clpXP*::*kanR*	pACYC-CpdRGFP-ChpT-CckA K- + pBAD-mCherry PopZ		this study

**GB#1974**	*E. coli* MG1655 DE3 *clpXP*::*kanR*	pACYC-CpdR$_{D51A}$ GFP-ChpT-CckA H+ + pBAD-mCherry PopZ		this study
**GB#1975**	*E. coli* MG1655 DE3 *clpXP*::*kanR*	pACYC-CpdRGFP-ChpT + pBAD-mCherry PopZ		this study
**GB#1976**	*E. coli* MG1655 DE3 *clpXP*::*kanR*	pACYC-CpdRGFP + pBAD-mCherry PopZ		this study
**GB#1977**	*E. coli* MG1655 DE3 *clpXP*::*kanR*	pACYC-CpdRGFP + pBAD-mCherry ΔN term PopZ		this study
**GB#1978**	*Caulobacter popZ::mChy-popZ*; GB#1007		pMCS5-pCpdR CpdR-YFP	this study
**GB#1979**	*Caulobacter popZ::mChy-popZ*; GB#1007		pMCS5-pCpdR CpdR$_{D51A}$-YFP	this study
**GB#1980**	*Caulobacter popZ::mChy-popZ*; GB#1007	pBXMCS2-CckA H+	pMCS5-pCpdR CpdR-YFP	this study
**GB#1981**	*Caulobacter popZ::mChy-popZ*; GB#1007	pBXMCS2-CckA K-	pMCS5-pCpdR CpdR-YFP	this study
**GB#1982**	*Caulobacter popZ::mChy-popZ*; GB#1007	pBXMCS2-CckA H+	pMCS5-pCpdR CpdR$_{D51A}$-YFP	this study
**GB#1983**	*Caulobacter* WT, GB#1194		pXMCS5-YFP-CtrA RD+15	this study
**GB#1984**	*Caulobacter* WT, GB#1194		pXMCS5-YFP-TacA	this study
**GB#1985**	*Caulobacter* WT, GB#1194		pXMCS5-YFP-PdeA	this study
**GB#1986**	*Caulobacter popZ::specR,* GB#1081		pXMCS5-YFP-CtrA RD+15	this study

**GB#1987**	*Caulobacter popZ::specR*, GB#1081		pXMCS5-YFP-TacA	this study
**GB#1988**	*Caulobacter popZ::specR*, GB#1081		pXMCS5-YFP-PdeA	this study
**GB#1989**	*Caulobacter* WT, GB#1194		pXMCS5-HA-CtrA RD+15	this study
**GB#1990**	*Caulobacter* WT, GB#1194		pXMCS5-HA-TacA	this study
**GB#1991**	*Caulobacter* WT, GB#1194		pXMCS5-HA-PdeA	this study
**GB#1992**	*Caulobacter popZ::specR*, GB#1081		pXMCS5-HA-CtrA RD+15	this study
**GB#1993**	*Caulobacter popZ::specR*, GB#1081		pXMCS5-HA-TacA	this study
**GB#1994**	*Caulobacter popZ::specR*, GB#1081		pXMCS5-HA-PdeA	this study
**GB#1995**	*Caulobacter cpdR::specR; popZ::mChy-popZ*			this study
**GB#1996**	*Caulobacter ΔrcdA, GB#1106*[8]		pMCS5-pCpdR CpdR-YFP	this study
**GB#1997**	*Caulobacter cpdR::specR, popZ::mChy-popZ;* GB#1995		pVMCS6-ClpX-msfGFP	this study
**GB#2002**	*Caulobacter cpdR::specR, popZ::mChy-popZ;* GB#1995		pVMCS6-RcdA-msfGFP	this study

**GB#2007**	*Caulobacter cpdR::specR, popZ::mChy-popZ;* GB#1995		pXMCS5-YFP-CtrA RD+15	this study
**GB#2008**	*Caulobacter cpdR::specR, popZ::mChy-popZ;* GB#1995		pXMCS5-YFP-TacA	this study
**GB#2009**	*Caulobacter cpdR::specR, popZ::mChy-popZ;* GB#1995		pXMCS5-YFP-PdeA	this study
**GB#2014**	*Caulobacter cpdR::specR, popZ::mChy-popZ;* GB#1995	pBXMCS2-pCpdR CpdR	pVMCS6-ClpX-GFP	this study
**GB#2015**	*Caulobacter cpdR::specR, popZ::mChy-popZ;* GB#1995	pBXMCS2-pCpdR $CpdR_{D51A}$	pVMCS6-ClpX-GFP	this study
**GB#2016**	*Caulobacter cpdR::specR, popZ::mChy-popZ;* GB#1995	pBXMCS2-pXyl-CckA H+ pCpdR CpdR	pVMCS6-ClpX-GFP	this study
**GB#2017**	*Caulobacter cpdR::specR, popZ::mChy-popZ;* GB#1995	pBXMCS2-pXyl-CckA H+ pCpdR $CpdR_{D51A}$	pVMCS6-ClpX-GFP	this study
**GB#2018**	*Caulobacter cpdR::specR, popZ::mChy-popZ;* GB#1995	pBXMCS2-pCpdR CpdR	pVMCS6-RcdA-GFP	this study
**GB#2019**	*Caulobacter cpdR::specR, popZ::mChy-popZ;* GB#1995	pBXMCS2-pCpdR $CpdR_{D51A}$	pVMCS6-RcdA-GFP	this study
**GB#2020**	*Caulobacter cpdR::specR,*	pBXMCS2-pXyl-CckA H+ pCpdR CpdR	pVMCS6-RcdA-GFP	this study

	popZ::mChy-popZ; GB#1995			
**GB#2021**	*Caulobacter cpdR::specR, popZ::mChy-popZ;* GB#1995	pBXMCS2-pXyl-CckA H+ pCpdR $CpdR_{D51A}$	pVMCS6-RcdA-GFP	this study

Supplementary Table 2

Plasmid name	Vector backbone	Description	Sequence<br>*where present, ellipsis correspond to vector backbone sequence
**pGB1945**	pCDF-duet	pCDF-CckA WT	gagatataccATGGCCGACTTGCAGCTCCAGGACAAGGTTTCGACCGGCGCCCCGCGTCGGCGGTTT<br>GATCCATGGCTGGTCGGCGCGGCGGTGTTTTTCGTGGCTGCGGCGGCCCTCTCGGCGGCGCCGG<br>CGCTCAAGGCCGGACCGACCACCCTGGCCGGCCTGCTGCTGCTTCTGGGCGTCGCGGGCGTGGC<br>TGTGTTGGGCCTTGTCGCCATTCGCGGCTCAGCGCTTTCCGGCGGCGACGCCGACCAGGCTGAG<br>GGGTTCATCGAGGCGCTGGCCGAGCCGGCCGCCCTGGCCGCCGCCGACGGTCGCGTCCTGGCCG<br>CCAACGGGCCCTGGCGCGAAGTCATGGGCGAACAGCGCCGCCTGCCCAAGGGCGTGGCGGGCT<br>CCAGCCTGTTTGCGGCCCTGGTCCAGGCGCGCCAGGGGCAGATGGCCGAGGGCATGCTGAGCGC<br>TGGAGGAACCGACTATACCGCCAAGGTCTCGCGCCTTGCGGGCGGACGGCTGATGATCCGGCTTG<br>CGCCCATCGTTGTCGCTGAGCCGGTTGTGGAAGACGCGTCGCCGGCCCCGGTCGCCGAGCGCGC<br>CGCCCCGCCACCCAGCTCGCTGGACGCCTTCGCCGGAGCCTCGCCGTTCGGCGCGGCCCTGCTGG<br>AAGGCCTGGAGCCGTTCACCTCGCGGGTGCTGGAGACCAATCCGGCGCTGACCACGATGACCGG<br>CGCCAAGGCCGGTGTGCTGTTCGGCGATCTGATCGACGCCGCCTCGCGCGCCGAGGCCGAGACG<br>CGCCTGAACGAAGGCCGCGCCGGTCCCTACGAGGTGCGGTTGGCGCGCGATCCGTCGCGCATCG<br>CTCACCTTTACCTCTATCGCGCCGAAGGCCGCCTGGTGGCCTACATGATCGACGTGTCCGAGCAGA<br>AGCAGATCGAGCTGCAGCTGTCCCAGGCCCAGAAGATGCAGGCCATCGGCCAGCTGGCCGGCGG<br>CGTCGCGCACGACTTCAACAACCTCTTGACCGCCATCCAGCTGCGTCTGGACGAGTTGCTGCATCG<br>CCATCCCGTCGGTGATCCGTCGTACGAGGGTCTCAACGAGATCCGTCAGACGGGCGTGCGCGCCG<br>CCGACCTCGTGCGCAAGCTCTTGGCTTTCTCGCGCAAGCAGACCGTGCAGCGCGAGGTGCTGGAT<br>CTGGGCGAGCTGATCAGCGAGTTCGAGGTCTTGCTGCGCCGCCTGCTGCGCGAAGACGTCAAGC<br>TGATCACCGACTATGGCCGCGACCTGCCGCAGGTGCGCGCCGACAAGAGCCAGCTCGAGACGGC<br>GGTCATGAACCTGGCCGTCAACGCCCGCGACGCCGTGCGCGCGGCCAAGGGCGGCGGCGTCGT<br>GCGCATCCGCACCGCGCGCCTGACCCGCGACGAGGCGATCCAGCTGGGTTTCCCGGCCGCCGAC<br>GGCGACACGGCCTTCATTGAGGTCAGTGACGATGGTCCGGGCATTCCGCCCGACGTCATGGGCAA<br>GATCTTCGACCCGTTCTTCACCACCAAGCCGGTGGGCGAGGGTACGGGCCTAGGCCTAGCCACGG<br>TCTATGGCATCGTTAAGCAGAGCGACGGCTGGATTCACGTCCACAGCCGTCCGAACGAAGGCGCG<br>GCCTTCCGCATCTTCCTGCCGGTCTATGAAGCGCCCGCCGGCGCGGTCGCCGTCCAGGCCGTCGC<br>CGAGCCCGCCAAGCCGCGCGCCGCTCGCGACCTGTCGGGCGCCGGCCGCATCCTGTTCGTCGAG<br>GACGAGGACGCCGTGCGCAGCGTCGCCGCCCGCCTGCTGCGCGCCCGTGGCTACGAGGTGCTTG<br>AGGCGGCCGACGGCGAAGAGGCCCTGATCATCGCCGAGGAAAACGCCGGCACGATCGACCTCTT<br>GATCAGCGACGTGATCATGCCCGGCATCGACGGCCCGACCTTGCTGAAGAAGGCGCGTGGCTATC<br>TGGGGACCGCGCCGGTGATGTTCATCTCCGGCTATGCCGAGGCCGAGTTCAGCGACCTCTTGGAA<br>GGCGAGACGGGCGTGACCTTCCTCCCCAAGCCGATCGACATCAAGACCCTGGCCGAGCGCGTCA<br>AGCAGCAGCTGCAGGCGGCGTAGgcggccgcat
**pGB1946**	pCDF-duet	pCDF-CckA H+	gagatataccATGGCCGACTTGCAGCTCCAGGACAAGGTTTCGACCGGCGCCCCGCGTCGGCGGTTT<br>GATCCATGGCTGGTCGGCGCGGCGGTGTTTTTCGTGGCTGCGGCGGCCCTCTCGGCGGCGCCGG<br>CGCTCAAGGCCGGACCGACCACCCTGGCCGGCCTGCTGCTGCTTCTGGGCGTCGCGGGCGTGGC<br>TGTGTTGGGCCTTGTCGCCATTCGCGGCTCAGCGCTTTCCGGCGGCGACGCCGACCAGGCTGAG<br>GGGTTCATCGAGGCGCTGGCCGAGCCGGCCGCCCTGGCCGCCGCCGACGGTCGCGTCCTGGCCG<br>CCAACGGGCCCTGGCGCGAAGTCATGGGCGAACAGCGCCGCCTGCCCAAGGGCGTGGCGGGCT<br>CCAGCCTGTTTGCGGCCCTGGTCCAGGCGCGCCAGGGGCAGATGGCCGAGGGCATGCTGAGCGC<br>TGGAGGAACCGACTATACCGCCAAGGTCTCGCGCCTTGCGGGCGGACGGCTGATGATCCGGCTTG<br>CGCCCATCGTTGTCGCTGAGCCGGTTGTGGAAGACGCGTCGCCGGCCCCGGTCGCCGAGCGCGC<br>CGCCCCGCCACCCAGCTCGCTGGACGCCTTCGCCGGAGCCTCGCCGTTCGGCGCGGCCCTGCTGG<br>AAGGCCTGGAGCCGTTCACCTCGCGGGTGCTGGAGACCAATCCGGCGCTGACCACGATGACCGG<br>CGCCAAGGCCGGTGTGCTGTTCGGCGATCTGATCGACGCCGCCTCGCGCGCCGAGGCCGAGACG<br>CGCCTGAACGAAGGCCGCGCCGGTCCCTACGAGGTGCGGTTGGCGCGCGATCCGTCGCGCATCG<br>CTCACCTTTACCTCTATCGCGCCGAAGGCCGCCTGGTGGCCTACATGATCGACGTGTCCGAGCAGA<br>AGCAGATCGAGCTGCAGCTGTCCCAGGCCCAGAAGATGCAGGCCATCGGCCAGCTGGCCGGCGA<br>AGTCGCGCACGACTTCAACAACCTCTTGACCGCCATCCAGCTGCGTCTGGACGAGTTGCTGCATCG<br>CCATCCCGTCGGTGATCCGTCGTACGAGGGTCTCAACGAGATCCGTCAGACGGGCGTGCGCGCCG<br>CCGACCTCGTGCGCAAGCTCTTGGCTTTCTCGCGCAAGCAGACCGTGCAGCGCGAGGTGCTGGAT

			CTGGGCGAGCTGATCAGCGAGTTCGAGGTCTTGCTGCGCCGCCTGCTGCGCGAAGACGTCAAGC<br>TGATCACCGACTATGGCCGCGACCTGCCGCAGGTGCGCGCCGACAAGAGCCAGCTCGAGACGGC<br>GGTCATGAACCTGGCCGTCAACGCCCGCGACGCCGTGCGCGCGGCCAAGGGCGGCGGCGTCGT<br>GCGCATCCGCACCGCGCGCCTGACCCGCGACGAGGCGATCCAGCTGGGTTTCCCGGCCGCCGAC<br>GGCGACACGGCCTTCATTGAGGTCAGTGACGATGGTCCGGGCATTCCGCCCGACGTCATGGGCAA<br>GATCTTCGACCCGTTCTTCACCACCAAGCCGGTGGGCGAGGGTACGGGCCTAGGCCTAGCCACGG<br>TCTATGGCATCGTTAAGCAGAGCGACGGCTGGATTCACGTCCACAGCCGTCCGAACGAAGGCGCG<br>GCCTTCCGCATCTTCCTGCCGGTCTATGAAGCGCCCGCCGGCGCGGTCGCCGTCCAGGCCGTCGC<br>CGAGCCCGCCAAGCCGCGCGCCGCTCGCGACCTGTCGGGCGCCGGCCGCATCCTGTTCGTCGAG<br>GACGAGGACGCCGTGCGCAGCGTCGCCGCCCGCCTGCTGCGCGCCCGTGGCTACGAGGTGCTTG<br>AGGCGGCCGACGGCGAAGAGGCCCTGATCATCGCCGAGGAAAACGCCGGCACGATCGACCTCTT<br>GATCAGCGACGTGATCATGCCCGGCATCGACGGCCCGACCTTGCTGAAGAAGGCGCGTGGCTATC<br>TGGGGACCGCGCCGGTGATGTTCATCTCCGGCTATGCCGAGGCCGAGTTCAGCGACCTCTTGGAA<br>GGCGAGACGGGCGTGACCTTCCTCCCCAAGCCGATCGACATCAAGACCCTGGCCGAGCGCGTCA<br>AGCAGCAGCTGCAGGCGGCGTAGgcggccgcat
**pGB1947**	pCDF-duet	pCDF-CckA<br>K-	gagatataccATGGCCGACTTGCAGCTCCAGGACAAGGTTTCGACCGGCGCCCCGCGTCGGCGGTTT<br>GATCCATGGCTGGTCGGCGCGGCGGTGTTTTTCGTGGCTGCGGCGGCCCTCTCGGCGGCGCCGG<br>CGCTCAAGGCCGGACCGACCACCCTGGCCGGCCTGCTGCTGCTTCTGGGCGTCGCGGGCGTGGC<br>TGTGTTGGGCCTTGTCGCCATTCGCGGCTCAGCGCTTTCCGGCGGCGACGCCGACCAGGCTGAG<br>GGGTTCATCGAGGCGCTGGCCGAGCCGGCCGCCCTGGCCGCCGCCGACGGTCGCGTCCTGGCCG<br>CCAACGGGCCCTGGCGCGAAGTCATGGGCGAACAGCGCCGCCTGCCCAAGGGCGTGGCGGGCT<br>CCAGCCTGTTTGCGGCCCTGGTCCAGGCGCGCCAGGGGCAGATGGCCGAGGGCATGCTGAGCGC<br>TGGAGGAACCGACTATACCGCCAAGGTCTCGCGCCTTGCGGGCGGACGGCTGATGATCCGGCTTG<br>CGCCCATCGTTGTCGCTGAGCCGGTTGTGGAAGACGCGTCGCCGGCCCCGGTCGCCGAGCGCGC<br>CGCCCCGCCACCCAGCTCGCTGGACGCCTTCGCCGGAGCCTCGCCGTTCGGCGCGGCCCTGCTGG<br>AAGGCCTGGAGCCGTTCACCTCGCGGGTGCTGGAGACCAATCCGGCGCTGACCACGATGACCGG<br>CGCCAAGGCCGGTGTGCTGTTCGGCGATCTGATCGACGCCGCCTCGCGCGCCGAGGCCGAGACG<br>CGCCTGAACGAAGGCCGCGCCGGTCCCTACGAGGTGCGGTTGGCGCGCGATCCGTCGCGCATCG<br>CTCACCTTTACCTCTATCGCGCCGAAGGCCGCCTGGTGGCCTACATGATCGACGTGTCCGAGCAGA<br>AGCAGATCGAGCTGCAGCTGTCCCAGGCCCAGAAGATGCAGGCCATCGGCCAGCTGGCCGGCGG<br>CGTCGCGCACGACTTCAACAACCTCTTGACCGCCATCCAGCTGCGTCTGGACGAGTTGCTGCATCG<br>CCATCCCGTCGGTGATCCGTCGTACGAGGGTCTCAACGAGATCCGTCAGACGGGCGTGCGCGCCG<br>CCGACCTCGTGCGCAAGCTCTTGGCTTTCTCGCGCAAGCAGACCGTGCAGCGCGAGGTGCTGGAT<br>CTGGGCGAGCTGATCAGCGAGTTCGAGGTCTTGCTGCGCCGCCTGCTGCGCGAAGACGTCAAGC<br>TGATCACCGACTATGGCCGCGACCTGCCGCAGGTGCGCGCCGACAAGAGCCAGCTCGAGACGGC<br>GGTCATGAACCTGGCCGTCAACGCCCGCGACGCCGTGCGCGCGGCCAAGGGCGGCGGCGTCGT<br>GCGCATCCGCACCGCGCGCCTGACCCGCGACGAGGCGATCCAGCTGGGTTTCCCGGCCGCCGAC<br>GGCGACACGGCCTTCATTGAGGTCAGTGACGATGGTCCGGGCATTCCGCCCGACGTCATGGGCAA<br>GATCTTCGACCCGTTCTTCACCACCAAGCCGGTGGGCGAGGGTACGGGCCTAGGCCTAGCCACGG<br>TCTATGGCATCGTTAAGCAGAGCGACGGCTGGATTCACGTCCACAGCCGTCCGAACGAAGGCGCG<br>GCCTTCCGCATCTTCCTGCCGGTCTATGAAGCGCCCGCCGGCGCGGTCGCCGTCCAGGCCGTCGC<br>CGAGCCCGCCAAGCCGCGCGCCGCTCGCGACCTGTCGGGCGCCGGCCGCATCCTGTTCGTCGAG<br>GACGAGGACGCCGTGCGCAGCGTCGCCGCCCGCCTGCTGCGCGCCCGTGGCTACGAGGTGCTTG<br>AGGCGGCCGACGGCGAAGAGGCCCTGATCATCGCCGAGGAAAACGCCGGCACGATCGACCTCTT<br>GATCAGCGACGTGATCATGCCCGGCATCGACGGCCCGACCTTGCTGAAGAAGGCGCGTGGCTATC<br>TGGGGACCGCGCCGGTGATGTTCATCTCCGGCTATGCCGAGGCCGAGTTCAGCGACCTCTTGGAA<br>GGCGAGACGGGCGTGACCTTCCTCCCCAAGCCGATCGACATCAAGACCCTGGCCGAGCGCGTCA<br>AGCAGCAGCTGCAGGCGGCGTAGgcggccgcat

**pGB1948**	pACYC-duet	pACYC-CpdRGFP-ChpT-CckAWT	gagatataccGTGGCCCGCATCCTCCTCGCCGAAGACGATGATTCCCTGCGCGGCTTCCTGGCCCGCG<br>CGCTGGAACGCGCCGGCTTCGAAGTCCAGGCCTGCGCCGACGGCGAAGAGGCCGTCCAGCACCT<br>GGACCATCCCTGGGACCTGCTGCTGACCGACATCGTCATGCCCGGCATGGACGGCATCGAGGTGG<br>CCCGCCAGGCCGCCGCCCGCGACCCGTCCCTGCGCATCATGTTCATCACCGGCTTCGCCGCCGTGG<br>CCCTCTCGGCCCAGGACCGCGCGCCCGCCGGCGCCAAGGTGCTGTCCAAGCCCGTGCACCTGCG<br>CGACCTCGTCGCCGAGGTCGAAAAGATGATGGCGGCCCTCGAGAGCGGCGGCGGCGGCAGTAA<br>AGGTGAAGAACTGTTCACCGGTGTTGTTCCGATCCTGGTTGAACTGGATGGTGATGTTAACGGCC<br>ACAAATTCTCTGTTCGTGGTGAAGGTGAAGGTGATGCAACCAACGGTAAACTGACCCTGAAATTC<br>ATCTGCACTACCGGTAAACTGCCGGTTCCATGGCCGACTCTGGTGACTACCCTGACCTATGGTGTTC<br>AGTGTTTTTCTCGTTACCCGGATCACATGAAGCAGCATGATTTCTTCAAATCTGCAATGCCGGAAGG<br>TTATGTACAGGAGCGCACCATTTCTTTCAAAGACGATGGCACCTACAAAACCCGTGCAGAGGTTAA<br>ATTTGAAGGTGATACTCTGGTGAACCGTATTGAACTGAAAGGCATTGATTTCAAAGAGGACGGCA<br>ACATCCTGGGCCACAAACTGGAATATAACTTCAACTCCCATAACGTTTACATCACCGCAGACAAACA<br>GAAGAACGGTATCAAAGCTAACTTCAAAATTCGCCATAACGTTGAAGACGGTAGCGTACAGCTGG<br>CGGACCACTACCAGCAGAACACTCCGATCGGTGATGGTCCGGTTCTGCTGCCGGATAACCACTACC<br>TGTCCACCCAGTCTAAACTGTCCAAAGACCCGAACGAAAAGCGCGACCACATGGTGCTGCTGGAG<br>TTCGTTACTGCAGCAGGTATCACGCACGGCATGGATGAACTCTACAAATGAgcggccgcat.........agat<br>atacatGTGACCGAGACCGTCACCGAGACCACCGCCCCCGCGTCCCCCGAAGCCGACGTCCAGGGT<br>CCCGATTTCGCCGCCATGCTGGCCGCGCGCCTGTGTCACGACTTCATCAGTCCGGCCAGCGCCATC<br>GTCTCGGGCCTGGATCTGCTGGAAGACCCCTCGGCCCAGGACATGCGCGACGACGCCATGAACCT<br>GATCGCCTCCTCGGCCCGCAAGCTGGCGGACCTCTTGCAGTTCACCCGAGTGGCCTTCGGCGCCT<br>CGGCCTCGGCCGAGAACTTCGACTCGCGCGAACTGGAAAAGCTGGCCCAGGGCGTCTTTGCCCA<br>TGTCCGCCCGACCCTGGACTGGCAGATCGAGCCGCAGGCGATGAACAAGCCCTCGTCGCGCGCG<br>GTGCTGAACATCGCCCAGATCGCCGCCAGCGCCCTGCCGGCCGGCGGCGTGGCCACCGTCAAGG<br>GCGTGGCCGCCGACGGGCGCTTCTCGATCATCGCCGACGCCAAGGGCCCGCGCGCGCGCCTGCG<br>TCCGGAGGTGCTGGCGGGCCTAAAGGGCGAGCCGCTGGCCGAGGGCCTGGGCGGTCCGTGGGT<br>GCAGGCGGCCTATCTGAACGCCCTGGTGCGCGCGGCCGGCGGCCAGATCGCCGTCGAGATCGGC<br>GAGGACCGCGCCTCGATCGCCGCCTGGGTCCCGGCGTAActcgagtctg.........tcagcgctagttatgcgac<br>tcctgcattaggaaattaatacgactcactataggggaattgtgagcggataacaattcccctgtagaaataattttgtttaacttt<br>aataaggagatataccATGGCCGACTTGCAGCTCCAGGACAAGGTTTCGACCGGCGCCCCGCGTCGGC<br>GGTTTGATCCATGGCTGGTCGGCGCGGCGGTGTTTTTCGTGGCTGCGGCGGCCCTCTCGGCGGCG<br>CCGGCGCTCAAGGCCGGACCGACCACCCTGGCCGGCCTGCTGCTGCTTCTGGGCGTCGCGGGCG<br>TGGCTGTGTTGGGCCTTGTCGCCATTCGCGGCTCAGCGCTTTCCGGCGGCGACGCCGACCAGGCT<br>GAGGGGTTCATCGAGGCGCTGGCCGAGCCGGCCGCCCTGGCCGCCGCCGACGGTCGCGTCCTGG<br>CCGCCAACGGGCCCTGGCGCGAAGTCATGGGCGAACAGCGCCGCCTGCCCAAGGGCGTGGCGG<br>GCTCCAGCCTGTTTGCGGCCCTGGTCCAGGCGCGCCAGGGGCAGATGGCCGAGGGCATGCTGAG<br>CGCTGGAGGAACCGACTATACCGCCAAGGTCTCGCGCCTTGCGGGCGGACGGCTGATGATCCGG<br>CTTGCGCCCATCGTTGTCGCTGAGCCGGTTGTGGAAGACGCGTCGCCGGCCCCGGTCGCCGAGC<br>GCGCCGCCCCGCCACCCAGCTCGCTGGACGCCTTCGCCGGAGCCTCGCCGTTCGGCGCGGCCCT<br>GCTGGAAGGCCTGGAGCCGTTCACCTCGCGGGTGCTGGAGACCAATCCGGCGCTGACCACGATG<br>ACCGGCGCCAAGGCCGGTGTGCTGTTCGGCGATCTGATCGACGCCGCCTCGCGCGCCGAGGCCG<br>AGACGCGCCTGAACGAAGGCCGCGCCGGTCCCTACGAGGTGCGGTTGGCGCGCGATCCGTCGCG<br>CATCGCTCACCTTTACCTCTATCGCGCCGAAGGCCGCCTGGTGGCCTACATGATCGACGTGTCCGA<br>GCAGAAGCAGATCGAGCTGCAGCTGTCCCAGGCCCAGAAGATGCAGGCCATCGGCCAGCTGGCC<br>GGCGGCGTCGCGCACGACTTCAACAACCTCTTGACCGCCATCCAGCTGCGTCTGGACGAGTTGCT<br>GCATCGCCATCCCGTCGGTGATCCGTCGTACGAGGGTCTCAACGAGATCCGTCAGACGGGCGTGC<br>GCGCCGCCGACCTCGTGCGCAAGCTCTTGGCTTTCTCGCGCAAGCAGACCGTGCAGCGCGAGGT<br>GCTGGATCTGGGCGAGCTGATCAGCGAGTTCGAGGTCTTGCTGCGCCGCCTGCTGCGCGAAGAC<br>GTCAAGCTGATCACCGACTATGGCCGCGACCTGCCGCAGGTGCGCGCCGACAAGAGCCAGCTCG<br>AGACGGCGGTCATGAACCTGGCCGTCAACGCCCGCGACGCCGTGCGCGCGGCCAAGGGCGGCG<br>GCGTCGTGCGCATCCGCACCGCGCGCCTGACCCGCGACGAGGCGATCCAGCTGGGTTTCCCGGCC<br>GCCGACGGCGACACGGCCTTCATTGAGGTCAGTGACGATGGTCCGGGCATTCCGCCCGACGTCAT<br>GGGCAAGATCTTCGACCCGTTCTTCACCACCAAGCCGGTGGGCGAGGGTACGGGCCTAGGCCTA<br>GCCACGGTCTATGGCATCGTTAAGCAGAGCGACGGCTGGATTCACGTCCACAGCCGTCCGAACGA<br>AGGCGCGGCCTTCCGCATCTTCCTGCCGGTCTATGAAGCGCCCGCCGGCGCGGTCGCCGTCCAGG

			CCGTCGCCGAGCCCGCCAAGCCGCGCGCCGCTCGCGACCTGTCGGGCGCCGGCCGCATCCTGTTC<br>GTCGAGGACGAGGACGCCGTGCGCAGCGTCGCCGCCCGCCTGCTGCGCGCCCGTGGCTACGAGG<br>TGCTTGAGGCGGCCGACGGCGAAGAGGCCCTGATCATCGCCGAGGAAAACGCCGGCACGATCGA<br>CCTCTTGATCAGCGACGTGATCATGCCCGGCATCGACGGCCCGACCTTGCTGAAGAAGGCGCGTG<br>GCTATCTGGGGACCGCGCCGGTGATGTTCATCTCCGGCTATGCCGAGGCCGAGTTCAGCGACCTCT<br>TGGAAGGCGAGACGGGCGTGACCTTCCTCCCCAAGCCGATCGACATCAAGACCCTGGCCGAGCG<br>CGTCAAGCAGCAGCTGCAGGCGGCGTAGcggagtgtat
**pGB1949**	pACYC-duet	pACYC-CpdRGFP-ChpT-CckA H+	gagatataccGTGGCCCGCATCCTCCTCGCCGAAGACGATGATTCCCTGCGCGGCTTCCTGGCCCGCG<br>CGCTGGAACGCGCCGGCTTCGAAGTCCAGGCCTGCGCCGACGGCGAAGAGGCCGTCCAGCACCT<br>GGACCATCCCTGGGACCTGCTGCTGACCGACATCGTCATGCCCGGCATGGACGGCATCGAGGTGG<br>CCCGCCAGGCCGCCGCCCGCGACCCGTCCCTGCGCATCATGTTCATCACCGGCTTCGCCGCCGTGG<br>CCCTCTCGGCCCAGGACCGCGCGCCCGCCGGCGCCAAGGTGCTGTCCAAGCCCGTGCACCTGCG<br>CGACCTCGTCGCCGAGGTCGAAAAGATGATGGCGGCCCTCGAGAGCGGCGGCGGCGGCAGTAA<br>AGGTGAAGAACTGTTCACCGGTGTTGTTCCGATCCTGGTTGAACTGGATGGTGATGTTAACGGCC<br>ACAAATTCTCTGTTCGTGGTGAAGGTGAAGGTGATGCAACCAACGGTAAACTGACCCTGAAATTC<br>ATCTGCACTACCGGTAAACTGCCGGTTCCATGGCCGACTCTGGTGACTACCCTGACCTATGGTGTTC<br>AGTGTTTTTCTCGTTACCCGGATCACATGAAGCAGCATGATTTCTTCAAATCTGCAATGCCGGAAGG<br>TTATGTACAGGAGCGCACCATTTCTTTCAAAGACGATGGCACCTACAAAACCCGTGCAGAGGTTAA<br>ATTTGAAGGTGATACTCTGGTGAACCGTATTGAACTGAAAGGCATTGATTTCAAAGAGGACGGCA<br>ACATCCTGGGCCACAAACTGGAATATAACTTCAACTCCCATAACGTTTACATCACCGCAGACAAACA<br>GAAGAACGGTATCAAAGCTAACTTCAAAATTCGCCATAACGTTGAAGACGGTAGCGTACAGCTGG<br>CGGACCACTACCAGCAGAACACTCCGATCGGTGATGGTCCGGTTCTGCTGCCGGATAACCACTACC<br>TGTCCACCCAGTCTAAACTGTCCAAAGACCCGAACGAAAAGCGCGACCACATGGTGCTGCTGGAG<br>TTCGTTACTGCAGCAGGTATCACGCACGGCATGGATGAACTCTACAAATGAgcggccgcat.........agat<br>atacatGTGACCGAGACCGTCACCGAGACCACCGCCCCCGCGTCCCCCGAAGCCGACGTCCAGGGT<br>CCCGATTTCGCCGCCATGCTGGCCGCGCGCCTGTGTCACGACTTCATCAGTCCGGCCAGCGCCATC<br>GTCTCGGGCCTGGATCTGCTGGAAGACCCCTCGGCCCAGGACATGCGCGACGACGCCATGAACCT<br>GATCGCCTCCTCGGCCCGCAAGCTGGCGGACCTCTTGCAGTTCACCCGAGTGGCCTTCGGCGCCT<br>CGGCCTCGGCCGAGAACTTCGACTCGCGCGAACTGGAAAAGCTGGCCCAGGGCGTCTTTGCCCA<br>TGTCCGCCCGACCCTGGACTGGCAGATCGAGCCGCAGGCGATGAACAAGCCCTCGTCGCGCGCG<br>GTGCTGAACATCGCCCAGATCGCCGCCAGCGCCCTGCCGGCCGGCGGCGTGGCCACCGTCAAGG<br>GCGTGGCCGCCGACGGGCGCTTCTCGATCATCGCCGACGCCAAGGGCCCGCGCGCGCGCCTGCG<br>TCCGGAGGTGCTGGCGGGCCTAAAGGGCGAGCCGCTGGCCGAGGGCCTGGGCGGTCCGTGGGT<br>GCAGGCGGCCTATCTGAACGCCCTGGTGCGCGCGGCCGGCGGCCAGATCGCCGTCGAGATCGGC<br>GAGGACCGCGCCTCGATCGCCGCCTGGGTCCCGGCGTAActcgagtctg.........tcagcgctagttatgcgac<br>tcctgcattaggaaattaatacgactcactataggggaattgtgagcggataacaattcccctgtagaaataattttgtttaacttt<br>aataaggagatataccATGGCCGACTTGCAGCTCCAGGACAAGGTTTCGACCGGCGCCCCGCGTCGGC<br>GGTTTGATCCATGGCTGGTCGGCGCGGCGGTGTTTTTCGTGGCTGCGGCGGCCCTCTCGGCGGCG<br>CCGGCGCTCAAGGCCGGACCGACCACCCTGGCCGGCCTGCTGCTGCTTCTGGGCGTCGCGGGCG<br>TGGCTGTGTTGGGCCTTGTCGCCATTCGCGGCTCAGCGCTTTCCGGCGGCGACGCCGACCAGGCT<br>GAGGGGTTCATCGAGGCGCTGGCCGAGCCGGCCGCCCTGGCCGCCGCCGACGGTCGCGTCCTGG<br>CCGCCAACGGGCCCTGGCGCGAAGTCATGGGCGAACAGCGCCGCCTGCCCAAGGGCGTGGCGG<br>GCTCCAGCCTGTTTGCGGCCCTGGTCCAGGCGCGCCAGGGGCAGATGGCCGAGGGCATGCTGAG<br>CGCTGGAGGAACCGACTATACCGCCAAGGTCTCGCGCCTTGCGGGCGGACGGCTGATGATCCGG<br>CTTGCGCCCATCGTTGTCGCTGAGCCGGTTGTGGAAGACGCGTCGCCGGCCCCGGTCGCCGAGC<br>GCGCCGCCCCGCCACCCAGCTCGCTGGACGCCTTCGCCGGAGCCTCGCCGTTCGGCGCGGCCCT<br>GCTGGAAGGCCTGGAGCCGTTCACCTCGCGGGTGCTGGAGACCAATCCGGCGCTGACCACGATG<br>ACCGGCGCCAAGGCCGGTGTGCTGTTCGGCGATCTGATCGACGCCGCCTCGCGCGCCGAGGCCG<br>AGACGCGCCTGAACGAAGGCCGCGCCGGTCCCTACGAGGTGCGGTTGGCGCGCGATCCGTCGCG<br>CATCGCTCACCTTTACCTCTATCGCGCCGAAGGCCGCCTGGTGGCCTACATGATCGACGTGTCCGA<br>GCAGAAGCAGATCGAGCTGCAGCTGTCCCAGGCCCAGAAGATGCAGGCCATCGGCCAGCTGGCC

			GGCGAAGTCGCGCACGACTTCAACAACCTCTTGACCGCCATCCAGCTGCGTCTGGACGAGTTGCT<br>GCATCGCCATCCCGTCGGTGATCCGTCGTACGAGGGTCTCAACGAGATCCGTCAGACGGGCGTGC<br>GCGCCGCCGACCTCGTGCGCAAGCTCTTGGCTTTCTCGCGCAAGCAGACCGTGCAGCGCGAGGT<br>GCTGGATCTGGGCGAGCTGATCAGCGAGTTCGAGGTCTTGCTGCGCCGCCTGCTGCGCGAAGAC<br>GTCAAGCTGATCACCGACTATGGCCGCGACCTGCCGCAGGTGCGCGCCGACAAGAGCCAGCTCG<br>AGACGGCGGTCATGAACCTGGCCGTCAACGCCCGCGACGCCGTGCGCGCGGCCAAGGGCGGCG<br>GCGTCGTGCGCATCCGCACCGCGCGCCTGACCCGCGACGAGGCGATCCAGCTGGGTTTCCCGGCC<br>GCCGACGGCGACACGGCCTTCATTGAGGTCAGTGACGATGGTCCGGGCATTCCGCCCGACGTCAT<br>GGGCAAGATCTTCGACCCGTTCTTCACCACCAAGCCGGTGGGCGAGGGTACGGGCCTAGGCCTA<br>GCCACGGTCTATGGCATCGTTAAGCAGAGCGACGGCTGGATTCACGTCCACAGCCGTCCGAACGA<br>AGGCGCGGCCTTCCGCATCTTCCTGCCGGTCTATGAAGCGCCCGCCGGCGCGGTCGCCGTCCAGG<br>CCGTCGCCGAGCCCGCCAAGCCGCGCGCCGCTCGCGACCTGTCGGGCGCCGGCCGCATCCTGTTC<br>GTCGAGGACGAGGACGCCGTGCGCAGCGTCGCCGCCCGCCTGCTGCGCGCCCGTGGCTACGAGG<br>TGCTTGAGGCGGCCGACGGCGAAGAGGCCCTGATCATCGCCGAGGAAAACGCCGGCACGATCGA<br>CCTCTTGATCAGCGACGTGATCATGCCCGGCATCGACGGCCCGACCTTGCTGAAGAAGGCGCGTG<br>GCTATCTGGGGACCGCGCCGGTGATGTTCATCTCCGGCTATGCCGAGGCCGAGTTCAGCGACCTCT<br>TGGAAGGCGAGACGGGCGTGACCTTCCTCCCCAAGCCGATCGACATCAAGACCCTGGCCGAGCG<br>CGTCAAGCAGCAGCTGCAGGCGGCGTAGcggagtgtat
**pGB1950**	pACYC-duet	pACYC-CpdRGFP-ChpT-CckAK-	gagatataccGTGGCCCGCATCCTCCTCGCCGAAGACGATGATTCCCTGCGCGGCTTCCTGGCCCGCG<br>CGCTGGAACGCGCCGGCTTCGAAGTCCAGGCCTGCGCCGACGGCGAAGAGGCCGTCCAGCACCT<br>GGACCATCCCTGGGACCTGCTGCTGACCGACATCGTCATGCCCGGCATGGACGGCATCGAGGTGG<br>CCCGCCAGGCCGCCGCCCGCGACCCGTCCCTGCGCATCATGTTCATCACCGGCTTCGCCGCCGTGG<br>CCCTCTCGGCCCAGGACCGCGCGCCCGCCGGCGCCAAGGTGCTGTCCAAGCCCGTGCACCTGCG<br>CGACCTCGTCGCCGAGGTCGAAAAGATGATGGCGGCCCTCGAGAGCGGCGGCGGCGGCAGTAA<br>AGGTGAAGAACTGTTCACCGGTGTTGTTCCGATCCTGGTTGAACTGGATGGTGATGTTAACGGCC<br>ACAAATTCTCTGTTCGTGGTGAAGGTGAAGGTGATGCAACCAACGGTAAACTGACCCTGAAATTC<br>ATCTGCACTACCGGTAAACTGCCGGTTCCATGGCCGACTCTGGTGACTACCCTGACCTATGGTGTTC<br>AGTGTTTTTCTCGTTACCCGGATCACATGAAGCAGCATGATTTCTTCAAATCTGCAATGCCGGAAGG<br>TTATGTACAGGAGCGCACCATTTCTTTCAAAGACGATGGCACCTACAAAACCCGTGCAGAGGTTAA<br>ATTTGAAGGTGATACTCTGGTGAACCGTATTGAACTGAAAGGCATTGATTTCAAAGAGGACGGCA<br>ACATCCTGGGCCACAAACTGGAATATAACTTCAACTCCCATAACGTTTACATCACCGCAGACAAACA<br>GAAGAACGGTATCAAAGCTAACTTCAAAATTCGCCATAACGTTGAAGACGGTAGCGTACAGCTGG<br>CGGACCACTACCAGCAGAACACTCCGATCGGTGATGGTCCGGTTCTGCTGCCGGATAACCACTACC<br>TGTCCACCCAGTCTAAACTGTCCAAAGACCCGAACGAAAAGCGCGACCACATGGTGCTGCTGGAG<br>TTCGTTACTGCAGCAGGTATCACGCACGGCATGGATGAACTCTACAAATGAgcggccgcat.........agat<br>atacatGTGACCGAGACCGTCACCGAGACCACCGCCCCCGCGTCCCCCGAAGCCGACGTCCAGGGT<br>CCCGATTTCGCCGCCATGCTGGCCGCGCGCCTGTGTCACGACTTCATCAGTCCGGCCAGCGCCATC<br>GTCTCGGGCCTGGATCTGCTGGAAGACCCCTCGGCCCAGGACATGCGCGACGACGCCATGAACCT<br>GATCGCCTCCTCGGCCCGCAAGCTGGCGGACCTCTTGCAGTTCACCCGAGTGGCCTTCGGCGCCT<br>CGGCCTCGGCCGAGAACTTCGACTCGCGCGAACTGGAAAAGCTGGCCCAGGGCGTCTTTGCCCA<br>TGTCCGCCCGACCCTGGACTGGCAGATCGAGCCGCAGGCGATGAACAAGCCCTCGTCGCGCGCG<br>GTGCTGAACATCGCCCAGATCGCCGCCAGCGCCCTGCCGGCCGGCGGCGTGGCCACCGTCAAGG<br>GCGTGGCCGCCGACGGGCGCTTCTCGATCATCGCCGACGCCAAGGGCCCGCGCGCGCGCCTGCG<br>TCCGGAGGTGCTGGCGGGCCTAAAGGGCGAGCCGCTGGCCGAGGGCCTGGGCGGTCCGTGGGT<br>GCAGGCGGCCTATCTGAACGCCCTGGTGCGCGCGGCCGGCGGCCAGATCGCCGTCGAGATCGGC<br>GAGGACCGCGCCTCGATCGCCGCCTGGGTCCCGGCGTAActcgagtctg.........tcagcgctagttatgcgac<br>tcctgcattaggaaattaatacgactcactataggggaattgtgagcggataacaattcccctgtagaaataattttgtttaacttt<br>aataaggagatataccATGGCCGACTTGCAGCTCCAGGACAAGGTTTCGACCGGCGCCCCGCGTCGGC<br>GGTTTGATCCATGGCTGGTCGGCGCGGCGGTGTTTTTCGTGGCTGCGGCGGCCCTCTCGGCGGCG<br>CCGGCGCTCAAGGCCGGACCGACCACCCTGGCCGGCCTGCTGCTGCTTCTGGGCGTCGCGGGCG<br>TGGCTGTGTTGGGCCTTGTCGCCATTCGCGGCTCAGCGCTTTCCGGCGGCGACGCCGACCAGGCT

			GAGGGGTTCATCGAGGCGCTGGCCGAGCCGGCCGCCCTGGCCGCCGCCGACGGTCGCGTCCTGG<br>CCGCCAACGGGCCCTGGCGCGAAGTCATGGGCGAACAGCGCCGCCTGCCCAAGGGCGTGGCGG<br>GCTCCAGCCTGTTTGCGGCCCTGGTCCAGGCGCGCCAGGGGCAGATGGCCGAGGGCATGCTGAG<br>CGCTGGAGGAACCGACTATACCGCCAAGGTCTCGCGCCTTGCGGGCGGACGGCTGATGATCCGG<br>CTTGCGCCCATCGTTGTCGCTGAGCCGGTTGTGGAAGACGCGTCGCCGGCCCCGGTCGCCGAGC<br>GCGCCGCCCCGCCACCCAGCTCGCTGGACGCCTTCGCCGGAGCCTCGCCGTTCGGCGCGGCCCT<br>GCTGGAAGGCCTGGAGCCGTTCACCTCGCGGGTGCTGGAGACCAATCCGGCGCTGACCACGATG<br>ACCGGCGCCAAGGCCGGTGTGCTGTTCGGCGATCTGATCGACGCCGCCTCGCGCGCCGAGGCCG<br>AGACGCGCCTGAACGAAGGCCGCGCCGGTCCCTACGAGGTGCGGTTGGCGCGCGATCCGTCGCG<br>CATCGCTCACCTTTACCTCTATCGCGCCGAAGGCCGCCTGGTGGCCTACATGATCGACGTGTCCGA<br>GCAGAAGCAGATCGAGCTGCAGCTGTCCCAGGCCCAGAAGATGCAGGCCATCGGCCAGCTGGCC<br>GGCGGCGTCGCGGCGGACTTCAACAACCTCTTGACCGCCATCCAGCTGCGTCTGGACGAGTTGCT<br>GCATCGCCATCCCGTCGGTGATCCGTCGTACGAGGGTCTCAACGAGATCCGTCAGACGGGCGTGC<br>GCGCCGCCGACCTCGTGCGCAAGCTCTTGGCTTTCTCGCGCAAGCAGACCGTGCAGCGCGAGGT<br>GCTGGATCTGGGCGAGCTGATCAGCGAGTTCGAGGTCTTGCTGCGCCGCCTGCTGCGCGAAGAC<br>GTCAAGCTGATCACCGACTATGGCCGCGACCTGCCGCAGGTGCGCGCCGACAAGAGCCAGCTCG<br>AGACGGCGGTCATGAACCTGGCCGTCAACGCCCGCGACGCCGTGCGCGCGGCCAAGGGCGGCG<br>GCGTCGTGCGCATCCGCACCGCGCGCCTGACCCGCGACGAGGCGATCCAGCTGGGTTTCCCGGCC<br>GCCGACGGCGACACGGCCTTCATTGAGGTCAGTGACGATGGTCCGGGCATTCCGCCCGACGTCAT<br>GGGCAAGATCTTCGACCCGTTCTTCACCACCAAGCCGGTGGGCGAGGGTACGGGCCTAGGCCTA<br>GCCACGGTCTATGGCATCGTTAAGCAGAGCGACGGCTGGATTCACGTCCACAGCCGTCCGAACGA<br>AGGCGCGGCCTTCCGCATCTTCCTGCCGGTCTATGAAGCGCCCGCCGGCGCGGTCGCCGTCCAGG<br>CCGTCGCCGAGCCCGCCAAGCCGCGCGCCGCTCGCGACCTGTCGGGCGCCGGCCGCATCCTGTTC<br>GTCGAGGACGAGGACGCCGTGCGCAGCGTCGCCGCCCGCCTGCTGCGCGCCCGTGGCTACGAGG<br>TGCTTGAGGCGGCCGACGGCGAAGAGGCCCTGATCATCGCCGAGGAAAACGCCGGCACGATCGA<br>CCTCTTGATCAGCGACGTGATCATGCCCGGCATCGACGGCCCGACCTTGCTGAAGAAGGCGCGTG<br>GCTATCTGGGGACCGCGCCGGTGATGTTCATCTCCGGCTATGCCGAGGCCGAGTTCAGCGACCTCT<br>TGGAAGGCGAGACGGGCGTGACCTTCCTCCCCAAGCCGATCGACATCAAGACCCTGGCCGAGCG<br>CGTCAAGCAGCAGCTGCAGGCGGCGTAGcggagtgtat
**pGB1951**	pACYC-duet	pACYC-CpdR$_{D51A}$ GFP-ChpT-CckA H+	gagatataccATGGCCCGCATCCTCCTCGCCGAAGACGATGATTCCCTGCGCGGCTTCCTGGCCCGCG<br>CGCTGGAACGCGCCGGCTTCGAAGTCCAGGCCTGCGCCGACGGCGAAGAGGCCGTCCAGCACCT<br>GGACCATCCCTGGGACCTGCTGCTGACCGCCATCGTCATGCCCGGCATGGACGGCATCGAGGTGG<br>CCCGCCAGGCCGCCGCCCGCGACCCGTCCCTGCGCATCATGTTCATCACCGGCTTCGCCGCCGTGG<br>CCCTCTCGGCCCAGGACCGCGCGCCCGCCGGCGCCAAGGTGCTGTCCAAGCCCGTGCACCTGCG<br>CGACCTCGTCGCCGAGGTCGAAAAGATGATGGCGGCCCTCGAGAGCGGCGGCGGCGGCAGTAA<br>AGGTGAAGAACTGTTCACCGGTGTTGTTCCGATCCTGGTTGAACTGGATGGTGATGTTAACGGCC<br>ACAAATTCTCTGTTCGTGGTGAAGGTGAAGGTGATGCAACCAACGGTAAACTGACCCTGAAATTC<br>ATCTGCACTACCGGTAAACTGCCGGTTCCATGGCCGACTCTGGTGACTACCCTGACCTATGGTGTTC<br>AGTGTTTTTCTCGTTACCCGGATCACATGAAGCAGCATGATTTCTTCAAATCTGCAATGCCGGAAGG<br>TTATGTACAGGAGCGCACCATTTCTTTCAAAGACGATGGCACCTACAAAACCCGTGCAGAGGTTAA<br>ATTTGAAGGTGATACTCTGGTGAACCGTATTGAACTGAAAGGCATTGATTTCAAAGAGGACGGCA<br>ACATCCTGGGCCACAAACTGGAATATAACTTCAACTCCCATAACGTTTACATCACCGCAGACAAACA<br>GAAGAACGGTATCAAAGCTAACTTCAAAATTCGCCATAACGTTGAAGACGGTAGCGTACAGCTGG<br>CGGACCACTACCAGCAGAACACTCCGATCGGTGATGGTCCGGTTCTGCTGCCGGATAACCACTACC<br>TGTCCACCCAGTCTAAACTGTCCAAAGACCCGAACGAAAAGCGCGACCACATGGTGCTGCTGGAG<br>TTCGTTACTGCAGCAGGTATCACGCACGGCATGGATGAACTCTACAAATGAgcggccgcat.........agat<br>atacatGTGACCGAGACCGTCACCGAGACCACCGCCCCCGCGTCCCCCGAAGCCGACGTCCAGGGT<br>CCCGATTTCGCCGCCATGCTGGCCGCGCGCCTGTGTCACGACTTCATCAGTCCGGCCAGCGCCATC<br>GTCTCGGGCCTGGATCTGCTGGAAGACCCCTCGGCCCAGGACATGCGCGACGACGCCATGAACCT<br>GATCGCCTCCTCGGCCCGCAAGCTGGCGGACCTCTTGCAGTTCACCCGAGTGGCCTTCGGCGCCT<br>CGGCCTCGGCCGAGAACTTCGACTCGCGCGAACTGGAAAAGCTGGCCCAGGGCGTCTTTGCCCA<br>TGTCCGCCCGACCCTGGACTGGCAGATCGAGCCGCAGGCGATGAACAAGCCCTCGTCGCGCGCG<br>GTGCTGAACATCGCCCAGATCGCCGCCAGCGCCCTGCCGGCCGGCGGCGTGGCCACCGTCAAGG<br>GCGTGGCCGCCGACGGGCGCTTCTCGATCATCGCCGACGCCAAGGGCCCGCGCGCGCGCCTGCG<br>TCCGGAGGTGCTGGCGGGCCTAAAGGGCGAGCCGCTGGCCGAGGGCCTGGGCGGTCCGTGGGT

			GCAGGCGGCCTATCTGAACGCCCTGGTGCGCGCGGCCGGCGGCCAGATCGCCGTCGAGATCGGC<br>GAGGACCGCGCCTCGATCGCCGCCTGGGTCCCGGCGTAActcgagtctg.........tcagcgctagttatgcgac<br>tcctgcattaggaaattaatacgactcactataggggaattgtgagcggataacaattcccctgtagaaataattttgtttaacttt<br>aataaggagatataccATGGCCGACTTGCAGCTCCAGGACAAGGTTTCGACCGGCGCCCCGCGTCGGC<br>GGTTTGATCCATGGCTGGTCGGCGCGGCGGTGTTTTTCGTGGCTGCGGCGGCCCTCTCGGCGGCG<br>CCGGCGCTCAAGGCCGGACCGACCACCCTGGCCGGCCTGCTGCTGCTTCTGGGCGTCGCGGGCG<br>TGGCTGTGTTGGGCCTTGTCGCCATTCGCGGCTCAGCGCTTTCCGGCGGCGACGCCGACCAGGCT<br>GAGGGGTTCATCGAGGCGCTGGCCGAGCCGGCCGCCCTGGCCGCCGCCGACGGTCGCGTCCTGG<br>CCGCCAACGGGCCCTGGCGCGAAGTCATGGGCGAACAGCGCCGCCTGCCCAAGGGCGTGGCGG<br>GCTCCAGCCTGTTTGCGGCCCTGGTCCAGGCGCGCCAGGGGCAGATGGCCGAGGGCATGCTGAG<br>CGCTGGAGGAACCGACTATACCGCCAAGGTCTCGCGCCTTGCGGGCGGACGGCTGATGATCCGG<br>CTTGCGCCCATCGTTGTCGCTGAGCCGGTTGTGGAAGACGCGTCGCCGGCCCCGGTCGCCGAGC<br>GCGCCGCCCCGCCACCCAGCTCGCTGGACGCCTTCGCCGGAGCCTCGCCGTTCGGCGCGGCCCT<br>GCTGGAAGGCCTGGAGCCGTTCACCTCGCGGGTGCTGGAGACCAATCCGGCGCTGACCACGATG<br>ACCGGCGCCAAGGCCGGTGTGCTGTTCGGCGATCTGATCGACGCCGCCTCGCGCGCCGAGGCCG<br>AGACGCGCCTGAACGAAGGCCGCGCCGGTCCCTACGAGGTGCGGTTGGCGCGCGATCCGTCGCG<br>CATCGCTCACCTTTACCTCTATCGCGCCGAAGGCCGCCTGGTGGCCTACATGATCGACGTGTCCGA<br>GCAGAAGCAGATCGAGCTGCAGCTGTCCCAGGCCCAGAAGATGCAGGCCATCGGCCAGCTGGCC<br>GGCGAAGTCGCGCACGACTTCAACAACCTCTTGACCGCCATCCAGCTGCGTCTGGACGAGTTGCT<br>GCATCGCCATCCCGTCGGTGATCCGTCGTACGAGGGTCTCAACGAGATCCGTCAGACGGGCGTGC<br>GCGCCGCCGACCTCGTGCGCAAGCTCTTGGCTTTCTCGCGCAAGCAGACCGTGCAGCGCGAGGT<br>GCTGGATCTGGGCGAGCTGATCAGCGAGTTCGAGGTCTTGCTGCGCCGCCTGCTGCGCGAAGAC<br>GTCAAGCTGATCACCGACTATGGCCGCGACCTGCCGCAGGTGCGCGCCGACAAGAGCCAGCTCG<br>AGACGGCGGTCATGAACCTGGCCGTCAACGCCCGCGACGCCGTGCGCGCGGCCAAGGGCGGCG<br>GCGTCGTGCGCATCCGCACCGCGCGCCTGACCCGCGACGAGGCGATCCAGCTGGGTTTCCCGGCC<br>GCCGACGGCGACACGGCCTTCATTGAGGTCAGTGACGATGGTCCGGGCATTCCGCCCGACGTCAT<br>GGGCAAGATCTTCGACCCGTTCTTCACCACCAAGCCGGTGGGCGAGGGTACGGGCCTAGGCCTA<br>GCCACGGTCTATGGCATCGTTAAGCAGAGCGACGGCTGGATTCACGTCCACAGCCGTCCGAACGA<br>AGGCGCGGCCTTCCGCATCTTCCTGCCGGTCTATGAAGCGCCCGCCGGCGCGGTCGCCGTCCAGG<br>CCGTCGCCGAGCCCGCCAAGCCGCGCGCCGCTCGCGACCTGTCGGGCGCCGGCCGCATCCTGTTC<br>GTCGAGGACGAGGACGCCGTGCGCAGCGTCGCCGCCCGCCTGCTGCGCGCCCGTGGCTACGAGG<br>TGCTTGAGGCGGCCGACGGCGAAGAGGCCCTGATCATCGCCGAGGAAAACGCCGGCACGATCGA<br>CCTCTTGATCAGCGACGTGATCATGCCCGGCATCGACGGCCCGACCTTGCTGAAGAAGGCGCGTG<br>GCTATCTGGGGACCGCGCCGGTGATGTTCATCTCCGGCTATGCCGAGGCCGAGTTCAGCGACCTCT<br>TGGAAGGCGAGACGGGCGTGACCTTCCTCCCCAAGCCGATCGACATCAAGACCCTGGCCGAGCG<br>CGTCAAGCAGCAGCTGCAGGCGGCGTAGcggagtgtat
**pGB1952**	pACYC-duet	pACYC-CpdRGFP-ChpT	gagatataccGTGGCCCGCATCCTCCTCGCCGAAGACGATGATTCCCTGCGCGGCTTCCTGGCCCGCG<br>CGCTGGAACGCGCCGGCTTCGAAGTCCAGGCCTGCGCCGACGGCGAAGAGGCCGTCCAGCACCT<br>GGACCATCCCTGGGACCTGCTGCTGACCGACATCGTCATGCCCGGCATGGACGGCATCGAGGTGG<br>CCCGCCAGGCCGCCGCCCGCGACCCGTCCCTGCGCATCATGTTCATCACCGGCTTCGCCGCCGTGG<br>CCCTCTCGGCCCAGGACCGCGCGCCCGCCGGCGCCAAGGTGCTGTCCAAGCCCGTGCACCTGCG<br>CGACCTCGTCGCCGAGGTCGAAAAGATGATGGCGGCCCTCGAGAGCGGCGGCGGCGGCAGTAA<br>AGGTGAAGAACTGTTCACCGGTGTTGTTCCGATCCTGGTTGAACTGGATGGTGATGTTAACGGCC<br>ACAAATTCTCTGTTCGTGGTGAAGGTGAAGGTGATGCAACCAACGGTAAACTGACCCTGAAATTC<br>ATCTGCACTACCGGTAAACTGCCGGTTCCATGGCCGACTCTGGTGACTACCCTGACCTATGGTGTTC<br>AGTGTTTTTCTCGTTACCCGGATCACATGAAGCAGCATGATTTCTTCAAATCTGCAATGCCGGAAGG<br>TTATGTACAGGAGCGCACCATTTCTTTCAAAGACGATGGCACCTACAAAACCCGTGCAGAGGTTAA<br>ATTTGAAGGTGATACTCTGGTGAACCGTATTGAACTGAAAGGCATTGATTTCAAAGAGGACGGCA<br>ACATCCTGGGCCACAAACTGGAATATAACTTCAACTCCCATAACGTTTACATCACCGCAGACAAACA<br>GAAGAACGGTATCAAAGCTAACTTCAAAATTCGCCATAACGTTGAAGACGGTAGCGTACAGCTGG<br>CGGACCACTACCAGCAGAACACTCCGATCGGTGATGGTCCGGTTCTGCTGCCGGATAACCACTACC<br>TGTCCACCCAGTCTAAACTGTCCAAAGACCCGAACGAAAAGCGCGACCACATGGTGCTGCTGGAG<br>TTCGTTACTGCAGCAGGTATCACGCACGGCATGGATGAACTCTACAAATGAgcggccgcat.........agat<br>atacatGTGACCGAGACCGTCACCGAGACCACCGCCCCCGCGTCCCCCGAAGCCGACGTCCAGGGT<br>CCCGATTTCGCCGCCATGCTGGCCGCGCGCCTGTGTCACGACTTCATCAGTCCGGCCAGCGCCATC

			GTCTCGGGCCTGGATCTGCTGGAAGACCCCTCGGCCCAGGACATGCGCGACGACGCCATGAACCT<br>GATCGCCTCCTCGGCCCGCAAGCTGGCGGACCTCTTGCAGTTCACCCGAGTGGCCTTCGGCGCCT<br>CGGCCTCGGCCGAGAACTTCGACTCGCGCGAACTGGAAAAGCTGGCCCAGGGCGTCTTTGCCCA<br>TGTCCGCCCGACCCTGGACTGGCAGATCGAGCCGCAGGCGATGAACAAGCCCTCGTCGCGCGCG<br>GTGCTGAACATCGCCCAGATCGCCGCCAGCGCCCTGCCGGCCGGCGGCGTGGCCACCGTCAAGG<br>GCGTGGCCGCCGACGGGCGCTTCTCGATCATCGCCGACGCCAAGGGCCCGCGCGCGCGCCTGCG<br>TCCGGAGGTGCTGGCGGGCCTAAAGGGCGAGCCGCTGGCCGAGGGCCTGGGCGGTCCGTGGGT<br>GCAGGCGGCCTATCTGAACGCCCTGGTGCGCGCGGCCGGCGGCCAGATCGCCGTCGAGATCGGC<br>GAGGACCGCGCCTCGATCGCCGCCTGGGTCCCGGCGTAActcgagtctg
**pGB1953**	pACYC-duet	pACYC-CpdRGFP	gagatataccGTGGCCCGCATCCTCCTCGCCGAAGACGATGATTCCCTGCGCGGCTTCCTGGCCCGCG<br>CGCTGGAACGCGCCGGCTTCGAAGTCCAGGCCTGCGCCGACGGCGAAGAGGCCGTCCAGCACCT<br>GGACCATCCCTGGGACCTGCTGCTGACCGACATCGTCATGCCCGGCATGGACGGCATCGAGGTGG<br>CCCGCCAGGCCGCCGCCCGCGACCCGTCCCTGCGCATCATGTTCATCACCGGCTTCGCCGCCGTGG<br>CCCTCTCGGCCCAGGACCGCGCGCCCGCCGGCGCCAAGGTGCTGTCCAAGCCCGTGCACCTGCG<br>CGACCTCGTCGCCGAGGTCGAAAAGATGATGGCGGCCCTCGAGAGCGGCGGCGGCGGCAGTAA<br>AGGTGAAGAACTGTTCACCGGTGTTGTTCCGATCCTGGTTGAACTGGATGGTGATGTTAACGGCC<br>ACAAATTCTCTGTTCGTGGTGAAGGTGAAGGTGATGCAACCAACGGTAAACTGACCCTGAAATTC<br>ATCTGCACTACCGGTAAACTGCCGGTTCCATGGCCGACTCTGGTGACTACCCTGACCTATGGTGTTC<br>AGTGTTTTTCTCGTTACCCGGATCACATGAAGCAGCATGATTTCTTCAAATCTGCAATGCCGGAAGG<br>TTATGTACAGGAGCGCACCATTTCTTTCAAAGACGATGGCACCTACAAAACCCGTGCAGAGGTTAA<br>ATTTGAAGGTGATACTCTGGTGAACCGTATTGAACTGAAAGGCATTGATTTCAAAGAGGACGGCA<br>ACATCCTGGGCCACAAACTGGAATATAACTTCAACTCCCATAACGTTTACATCACCGCAGACAAACA<br>GAAGAACGGTATCAAAGCTAACTTCAAAATTCGCCATAACGTTGAAGACGGTAGCGTACAGCTGG<br>CGGACCACTACCAGCAGAACACTCCGATCGGTGATGGTCCGGTTCTGCTGCCGGATAACCACTACC<br>TGTCCACCCAGTCTAAACTGTCCAAAGACCCGAACGAAAAGCGCGACCACATGGTGCTGCTGGAG<br>TTCGTTACTGCAGCAGGTATCACGCACGGCATGGATGAACTCTACAAATGAgcggccgcat
**pGB1954**	pBAD	pBAD-mCherry ΔN term PopZ	ggaattaaccATGGTGAGCAAGGGCGAGGAGGATAACATGGCCATCATCAAGGAGTTCATGCGCTTC<br>AAGGTGCACATGGAGGGCTCCGTGAACGGCCACGAGTTCGAGATCGAGGGCGAGGGCGAGGGC<br>CGCCCCTACGAGGGCACCCAGACCGCCAAGCTGAAGGTGACCAAGGGTGGCCCCCTGCCCTTCG<br>CCTGGGACATCCTGTCCCCTCAGTTCATGTACGGCTCCAAGGCCTACGTGAAGCACCCCGCCGACA<br>TCCCCGACTACTTGAAGCTGTCCTTCCCCGAGGGCTTCAAGTGGGAGCGCGTGATGAACTTCGAG<br>GACGGCGGCGTGGTGACCGTGACCCAGGACTCCTCCCTGCAGGACGGCGAGTTCATCTACAAGG<br>TGAAGCTGCGCGGCACCAACTTCCCCTCCGACGGCCCCGTAATGCAGAAGAAGACCATGGGCTG<br>GGAGGCCTCCTCCGAGCGGATGTACCCCGAGGACGGCGCCCTGAAGGGCGAGATCAAGCAGAG<br>GCTGAAGCTGAAGGACGGCGGCCACTACGACGCTGAGGTCAAGACCACCTACAAGGCCAAGAA<br>GCCCGTGCAGCTGCCCGGCGCCTACAACGTCAACATCAAGTTGGACATCACCTCCCACAACGAGG<br>ACTACACCATCGTGGAACAGTACGAACGCGCCGAGGGCCGCCACTCCACCGGCGGCATGGACGA<br>GCTGTACAAGcctgcaggcgccttaattaatatgcatggtaccGATGACGCGCCGGCGGAGCCTGCGGCCGA<br>AGCGGCGCCCCCGCCGCCGCCGGAACCCGAACCTGAACCGGTGTCGTTCGACGACGAGGTTCTG<br>GAATTGACGGATCCGATCGCGCCCGAGCCCGAGCTGCCGCCGCTGGAGACTGTCGGCGACATCG<br>ACGTCTATTCGCCGCCGGAACCTGAGTCGGAACCGGCCTACACGCCGCCGCCGGCGGCTCCGGTG<br>TTTGATCGCGACGAAGTCGCCGAGCAGCTGGTCGGCGTTTCGGCCGCTTCGGCCGCGGCGAGCG<br>CCTTCGGCAGCCTGAGCTCGGCCCTGCTGATGCCCAAGGACGGTCGGACGCTGGAAGACGTCGT<br>ACGCGAGCTGCTGCGCCCGCTGCTCAAGGAGTGGCTGGACCAGAACCTGCCGCGCATCGTCGAG<br>ACCAAGGTTGAGGAAGAAGTGCAGCGTATCTCTCGGGGACGCGGCGCCTAActcgagatct
**pGB1955**	pMCS5 (NdeI, KpnI)	pMCS5-pCpdR CpdR YFP	ccaattgcatGGTCGCCGTCGAACATCGCCGGGTCCAGCTCCCAGGGCTCACGGTTCAGATCCACATA<br>GGCGCGGGCGAAGCGGGCGCGCACCACGGCGGCCCCCAGGGCGGGCGCGCCGCCGATGATGC<br>GGTCGACAAACGCGTCCTCGGACGCGCGCAGGGTCTCGAGCGGCAGGCGGACCGCGGCGACCA<br>TGTCCTCCGGATAGAGGTCGCCCGAGTGCGGCGAGGCGAACACCAAGGCGGTCGGCGGCGGCG<br>CCCCGGCCGGGACCGCGCGCAGCACCTCGAACGCTGGCCCGCCAAAGGTCTCCAGCGGCGGGG<br>ACGAAAGCTCTTCGCTCGAAAGGGGCCGCCACGCGCTCATGCCGCATCATCGAAGCGTCGCGGCG<br>CGGGGTCAAATCTCAATCGCCGCTGACGAAACATCCCCAGCCGCGACGTTTAGGTTCATCCCCGAT<br>TTACGGACGGGGCGATAAGGTGGATCCTCTATCGACGATCTTAATCGGACACGTGACCCCATGGCC<br>CGCATCCTCCTCGCCGAAGACGATGATTCCCTGCGCGGCTTCCTGGCCCGCGCGCTGGAACGCGC

			CGGCTTCGAAGTCCAGGCCTGCGCCGACGGCGAAGAGGCCGTCCAGCACCTGGACCATCCCTGG<br>GACCTGCTGCTGACCGACATCGTCATGCCCGGCATGGACGGCATCGAGGTGGCCCGCCAGGCCGC<br>CGCCCGCGACCCGTCCCTGCGCATCATGTTCATCACCGGCTTCGCCGCCGTGGCCCTCTCGGCCCA<br>GGACCGCGCGCCCGCCGGCGCCAAGGTGCTGTCCAAGCCCGTGCACCTGCGCGACCTCGTCGCC<br>GAGGTCGAAAAGATGATGGCGGCCCGGTACCGCGGGCCCGGGATCCACATGGTGAGCAAGGGC<br>GAGGAGCTGTTCACCGGGGTGGTGCCCATCCTGGTCGAGCTGGACGGCGACGTAAACGGCCACA<br>AGTTCAGCGTGTCCGGCGAGGGCGAGGGCGATGCCACCTACGGCAAGCTGACCCTGAAGTTCAT<br>CTGCACCACCGGCAAGCTGCCCGTGCCCTGGCCCACCCTCGTGACCACCTTCGGCTACGGCCTGC<br>AGTGCTTCGCCCGCTACCCCGACCACATGAAGCAGCACGACTTCTTCAAGTCCGCCATGCCCGAAG<br>GCTACGTCCAGGAGCGCACCATCTTCTTCAAGGACGACGGCAACTACAAGACCCGCGCCGAGGT<br>GAAGTTCGAGGGCGACACCCTGGTGAACCGCATCGAGCTGAAGGGCATCGACTTCAAGGAGGAC<br>GGCAACATCCTGGGGCACAAGCTGGAGTACAACTACAACAGCCACAACGTCTATATCATGGCCGA<br>CAAGCAGAAGAACGGCATCAAGGTGAACTTCAAGATCCGCCACAACATCGAGGACGGCAGCGTG<br>CAGCTCGCCGACCACTACCAGCAGAACACCCCCATCGGCGACGGCCCCGTGCTGCTGCCCGACAA<br>CCACTACCTGAGCTACCAGTCCGCCCTGAGCAAAGACCCCAACGAGAAGCGCGATCACATGGTCC<br>TGCTGGAGTTCGTGACCGCCGCCGGGATCACTCTCGGCATGGACGAGCTGTACAAGTAGggtacctt<br>aa
**pGB1956**	pMCS5	pMCS5-<br>pCpdR<br>$CpdR_{D51A}$<br>YFP	ccaattgcatGGTCGCCGTCGAACATCGCCGGGTCCAGCTCCCAGGGCTCACGGTTCAGATCCACATA<br>GGCGCGGGCGAAGCGGGCGCGCACCACGGCGGCCCCCAGGGCGGGCGCGCCGCCGATGATGC<br>GGTCGACAAACGCGTCCTCGGACGCGCGCAGGGTCTCGAGCGGCAGGCGGACCGCGGCGACCA<br>TGTCCTCCGGATAGAGGTCGCCCGAGTGCGGCGAGGCGAACACCAAGGCGGTCGGCGGCGGCG<br>CCCCGGCCGGGACCGCGCGCAGCACCTCGAACGCTGGCCCGCCAAAGGTCTCCAGCGGCGGGG<br>ACGAAAGCTCTTCGCTCGAAAGGGGCCGCCACGCGCTCATGCCGCATCATCGAAGCGTCGCGGCG<br>CGGGGTCAAATCTCAATCGCCGCTGACGAAACATCCCCAGCCGCGACGTTTAGGTTCATCCCCGAT<br>TTACGGACGGGGCGATAAGGTGGATCCTCTATCGACGATCTTAATCGGACACGTGACCCCATGGCC<br>CGCATCCTCCTCGCCGAAGACGATGATTCCCTGCGCGGCTTCCTGGCCCGCGCGCTGGAACGCGC<br>CGGCTTCGAAGTCCAGGCCTGCGCCGACGGCGAAGAGGCCGTCCAGCACCTGGACCATCCCTGG<br>GACCTGCTGCTGACCGCCATCGTCATGCCCGGCATGGACGGCATCGAGGTGGCCCGCCAGGCCGC<br>CGCCCGCGACCCGTCCCTGCGCATCATGTTCATCACCGGCTTCGCCGCCGTGGCCCTCTCGGCCCA<br>GGACCGCGCGCCCGCCGGCGCCAAGGTGCTGTCCAAGCCCGTGCACCTGCGCGACCTCGTCGCC<br>GAGGTCGAAAAGATGATGGCGGCCCGGTACCGCGGGCCCGGGATCCACATGGTGAGCAAGGGC<br>GAGGAGCTGTTCACCGGGGTGGTGCCCATCCTGGTCGAGCTGGACGGCGACGTAAACGGCCACA<br>AGTTCAGCGTGTCCGGCGAGGGCGAGGGCGATGCCACCTACGGCAAGCTGACCCTGAAGTTCAT<br>CTGCACCACCGGCAAGCTGCCCGTGCCCTGGCCCACCCTCGTGACCACCTTCGGCTACGGCCTGC<br>AGTGCTTCGCCCGCTACCCCGACCACATGAAGCAGCACGACTTCTTCAAGTCCGCCATGCCCGAAG<br>GCTACGTCCAGGAGCGCACCATCTTCTTCAAGGACGACGGCAACTACAAGACCCGCGCCGAGGT<br>GAAGTTCGAGGGCGACACCCTGGTGAACCGCATCGAGCTGAAGGGCATCGACTTCAAGGAGGAC<br>GGCAACATCCTGGGGCACAAGCTGGAGTACAACTACAACAGCCACAACGTCTATATCATGGCCGA<br>CAAGCAGAAGAACGGCATCAAGGTGAACTTCAAGATCCGCCACAACATCGAGGACGGCAGCGTG<br>CAGCTCGCCGACCACTACCAGCAGAACACCCCCATCGGCGACGGCCCCGTGCTGCTGCCCGACAA<br>CCACTACCTGAGCTACCAGTCCGCCCTGAGCAAAGACCCCAACGAGAAGCGCGATCACATGGTCC<br>TGCTGGAGTTCGTGACCGCCGCCGGGATCACTCTCGGCATGGACGAGCTGTACAAGTAGggtacctt<br>aa
**pGB1959**	pBXMCS2<br>(NdeI,<br>EcoRI)	pBXMCS2-<br>CckA H+	agacgaccatATGGCCGACTTGCAGCTCCAGGACAAGGTTTCGACCGGCGCCCCGCGTCGGCGGTTT<br>GATCCATGGCTGGTCGGCGCGGCGGTGTTTTTCGTGGCTGCGGCGGCCCTCTCGGCGGCGCCGG<br>CGCTCAAGGCCGGACCGACCACCCTGGCCGGCCTGCTGCTGCTTCTGGGCGTCGCGGGCGTGGC<br>TGTGTTGGGCCTTGTCGCCATTCGCGGCTCAGCGCTTTCCGGCGGCGACGCCGACCAGGCTGAG<br>GGGTTCATCGAGGCGCTGGCCGAGCCGGCCGCCCTGGCCGCCGCCGACGGTCGCGTCCTGGCCG<br>CCAACGGGCCCTGGCGCGAAGTCATGGGCGAACAGCGCCGCCTGCCCAAGGGCGTGGCGGGCT<br>CCAGCCTGTTTGCGGCCCTGGTCCAGGCGCGCCAGGGGCAGATGGCCGAGGGCATGCTGAGCGC<br>TGGAGGAACCGACTATACCGCCAAGGTCTCGCGCCTTGCGGGCGGACGGCTGATGATCCGGCTTG<br>CGCCCATCGTTGTCGCTGAGCCGGTTGTGGAAGACGCGTCGCCGGCCCCGGTCGCCGAGCGCGC<br>CGCCCCGCCACCCAGCTCGCTGGACGCCTTCGCCGGAGCCTCGCCGTTCGGCGCGGCCCTGCTGG<br>AAGGCCTGGAGCCGTTCACCTCGCGGGTGCTGGAGACCAATCCGGCGCTGACCACGATGACCGG<br>CGCCAAGGCCGGTGTGCTGTTCGGCGATCTGATCGACGCCGCCTCGCGCGCCGAGGCCGAGACG

			CGCCTGAACGAAGGCCGCGCCGGTCCCTACGAGGTGCGGTTGGCGCGCGATCCGTCGCGCATCG<br>CTCACCTTTACCTCTATCGCGCCGAAGGCCGCCTGGTGGCCTACATGATCGACGTGTCCGAGCAGA<br>AGCAGATCGAGCTGCAGCTGTCCCAGGCCCAGAAGATGCAGGCCATCGGCCAGCTGGCCGGCGA<br>AGTCGCGCACGACTTCAACAACCTCTTGACCGCCATCCAGCTGCGTCTGGACGAGTTGCTGCATCG<br>CCATCCCGTCGGTGATCCGTCGTACGAGGGTCTCAACGAGATCCGTCAGACGGGCGTGCGCGCCG<br>CCGACCTCGTGCGCAAGCTCTTGGCTTTCTCGCGCAAGCAGACCGTGCAGCGCGAGGTGCTGGAT<br>CTGGGCGAGCTGATCAGCGAGTTCGAGGTCTTGCTGCGCCGCCTGCTGCGCGAAGACGTCAAGC<br>TGATCACCGACTATGGCCGCGACCTGCCGCAGGTGCGCGCCGACAAGAGCCAGCTCGAGACGGC<br>GGTCATGAACCTGGCCGTCAACGCCCGCGACGCCGTGCGCGCGGCCAAGGGCGGCGGCGTCGT<br>GCGCATCCGCACCGCGCGCCTGACCCGCGACGAGGCGATCCAGCTGGGTTTCCCGGCCGCCGAC<br>GGCGACACGGCCTTCATTGAGGTCAGTGACGATGGTCCGGGCATTCCGCCCGACGTCATGGGCAA<br>GATCTTCGACCCGTTCTTCACCACCAAGCCGGTGGGCGAGGGTACGGGCCTAGGCCTAGCCACGG<br>TCTATGGCATCGTTAAGCAGAGCGACGGCTGGATTCACGTCCACAGCCGTCCGAACGAAGGCGCG<br>GCCTTCCGCATCTTCCTGCCGGTCTATGAAGCGCCCGCCGGCGCGGTCGCCGTCCAGGCCGTCGC<br>CGAGCCCGCCAAGCCGCGCGCCGCTCGCGACCTGTCGGGCGCCGGCCGCATCCTGTTCGTCGAG<br>GACGAGGACGCCGTGCGCAGCGTCGCCGCCCGCCTGCTGCGCGCCCGTGGCTACGAGGTGCTTG<br>AGGCGGCCGACGGCGAAGAGGCCCTGATCATCGCCGAGGAAAACGCCGGCACGATCGACCTCTT<br>GATCAGCGACGTGATCATGCCCGGCATCGACGGCCCGACCTTGCTGAAGAAGGCGCGTGGCTATC<br>TGGGGACCGCGCCGGTGATGTTCATCTCCGGCTATGCCGAGGCCGAGTTCAGCGACCTCTTGGAA<br>GGCGAGACGGGCGTGACCTTCCTCCCCAAGCCGATCGACATCAAGACCCTGGCCGAGCGCGGTC<br>AAGCAGCAGCTGCAAGCAGCATAGgaattcctgc
**pGB1960**	pBXMCS2	pBXMCS2-<br>CckA K-	agacgaccatATGGCCGACTTGCAGCTCCAGGACAAGGTTTCGACCGGCGCCCCGCGTCGGCGGTTT<br>GATCCATGGCTGGTCGGCGCGGCGGTGTTTTTCGTGGCTGCGGCGGCCCTCTCGGCGGCGCCGG<br>CGCTCAAGGCCGGACCGACCACCCTGGCCGGCCTGCTGCTGCTTCTGGGCGTCGCGGGCGTGGC<br>TGTGTTGGGCCTTGTCGCCATTCGCGGCTCAGCGCTTTCCGGCGGCGACGCCGACCAGGCTGAG<br>GGGTTCATCGAGGCGCTGGCCGAGCCGGCCGCCCTGGCCGCCGCCGACGGTCGCGTCCTGGCCG<br>CCAACGGGCCCTGGCGCGAAGTCATGGGCGAACAGCGCCGCCTGCCCAAGGGCGTGGCGGGCT<br>CCAGCCTGTTTGCGGCCCTGGTCCAGGCGCGCCAGGGGCAGATGGCCGAGGGCATGCTGAGCGC<br>TGGAGGAACCGACTATACCGCCAAGGTCTCGCGCCTTGCGGGCGGACGGCTGATGATCCGGCTTG<br>CGCCCATCGTTGTCGCTGAGCCGGTTGTGGAAGACGCGTCGCCGGCCCCGGTCGCCGAGCGCGC<br>CGCCCCGCCACCCAGCTCGCTGGACGCCTTCGCCGGAGCCTCGCCGTTCGGCGCGGCCCTGCTGG<br>AAGGCCTGGAGCCGTTCACCTCGCGGGTGCTGGAGACCAATCCGGCGCTGACCACGATGACCGG<br>CGCCAAGGCCGGTGTGCTGTTCGGCGATCTGATCGACGCCGCCTCGCGCGCCGAGGCCGAGACG<br>CGCCTGAACGAAGGCCGCGCCGGTCCCTACGAGGTGCGGTTGGCGCGCGATCCGTCGCGCATCG<br>CTCACCTTTACCTCTATCGCGCCGAAGGCCGCCTGGTGGCCTACATGATCGACGTGTCCGAGCAGA<br>AGCAGATCGAGCTGCAGCTGTCCCAGGCCCAGAAGATGCAGGCCATCGGCCAGCTGGCCGGCGG<br>CGTCGCGGCGGACTTCAACAACCTCTTGACCGCCATCCAGCTGCGTCTGGACGAGTTGCTGCATC<br>GCCATCCCGTCGGTGATCCGTCGTACGAGGGTCTCAACGAGATCCGTCAGACGGGCGTGCGCGCC<br>GCCGACCTCGTGCGCAAGCTCTTGGCTTTCTCGCGCAAGCAGACCGTGCAGCGCGAGGTGCTGG<br>ATCTGGGCGAGCTGATCAGCGAGTTCGAGGTCTTGCTGCGCCGCCTGCTGCGCGAAGACGTCAA<br>GCTGATCACCGACTATGGCCGCGACCTGCCGCAGGTGCGCGCCGACAAGAGCCAGCTCGAGACG<br>GCGGTCATGAACCTGGCCGTCAACGCCCGCGACGCCGTGCGCGCGGCCAAGGGCGGCGGCGTC<br>GTGCGCATCCGCACCGCGCGCCTGACCCGCGACGAGGCGATCCAGCTGGGTTTCCCGGCCGCCG<br>ACGGCGACACGGCCTTCATTGAGGTCAGTGACGATGGTCCGGGCATTCCGCCCGACGTCATGGGC<br>AAGATCTTCGACCCGTTCTTCACCACCAAGCCGGTGGGCGAGGGTACGGGCCTAGGCCTAGCCAC<br>GGTCTATGGCATCGTTAAGCAGAGCGACGGCTGGATTCACGTCCACAGCCGTCCGAACGAAGGCG<br>CGGCCTTCCGCATCTTCCTGCCGGTCTATGAAGCGCCCGCCGGCGCGGTCGCCGTCCAGGCCGTC<br>GCCGAGCCCGCCAAGCCGCGCGCCGCTCGCGACCTGTCGGGCGCCGGCCGCATCCTGTTCGTCG<br>AGGACGAGGACGCCGTGCGCAGCGTCGCCGCCCGCCTGCTGCGCGCCCGTGGCTACGAGGTGCT<br>TGAGGCGGCCGACGGCGAAGAGGCCCTGATCATCGCCGAGGAAAACGCCGGCACGATCGACCTC<br>TTGATCAGCGACGTGATCATGCCCGGCATCGACGGCCCGACCTTGCTGAAGAAGGCGCGTGGCTA<br>TCTGGGGACCGCGCCGGTGATGTTCATCTCCGGCTATGCCGAGGCCGAGTTCAGCGACCTCTTGG<br>AAGGCGAGACGGGCGTGACCTTCCTCCCCAAGCCGATCGACATCAAGACCCTGGCCGAGCGCGT<br>CAAGCAGCAGCTGCAAGCAGCATAGgaattcctgc

**pGB1961**	pXMCS5 (NdeI, KpnI)	pXMCS5-YFP-CtrA RD+15	agacgaccatATGAGCAAGGGCGAGGAGCTGTTCACCGGGGTGGTGCCCATCCTGGTCGAGCTGGACGGCGACGTAAACGGCCACAAGTTCAGCGTGTCCGGCGAGGGCGAGGGCGATGCCACCTACGGCAAGCTGACCCTGAAGTTCATCTGCACCACCGGCAAGCTGCCCGTGCCCTGGCCCACCCTCGTGACCACCTTCGGCTACGGCCTGCAGTGCTTCGCCCGCTACCCCGACCACATGAAGCAGCACGACTTCTTCAAGTCCGCCATGCCCGAAGGCTACGTCCAGGAGCGCACCATCTTCTTCAAGGACGACGGCAACTACAAGACCCGCGCCGAGGTGAAGTTCGAGGGCGACACCCTGGTGAACCGCATCGAGCTGAAGGGCATCGACTTCAAGGAGGACGGCAACATCCTGGGGCACAAGCTGGAGTACAACTACAACAGCCACAACGTCTATATCATGGCCGACAAGCAGAAGAACGGCATCAAGGTGAACTTCAAGATCCGCCACAACATCGAGGACGGCAGCGTGCAGCTCGCCGACCACTACCAGCAGAACACCCCCATCGGCGACGGCCCCGTGCTGCTGCCCGACAACCACTACCTGAGCTACCAGTCCGCCCTGAGCAAAGACCCCAACGAGAAGCGCGATCACATGGTCCTGCTGGAGTTCGTGACCGCCGCCGGGATCACTCTCGGCATGGACGAGCTGTACAAGggaagtagcggtGCgCGCGTACTGTTGATCGAGGATGACAGCGCGACGGCGCAGACCATCGAACTGATGCTGAAGTCTGAAGGCTTCAACGTCTATACGACGGATCTGGGTGAAGAAGGCGTCGATCTGGGCAAGATCTACGACTACGATCTTATCCTGCTCGACCTCAATCTTCCGGACATGAGCGGCATCGATGTTCTGCGCACCCTGCGGGTCGCGAAGATCAACACGCCCATCATGATCCTGTCGGGCTCGTCGGAAATCGACACCAAGGTCAAGACCTTCGCCGGCGGCGCCGACGACTACATGACCAAGCCGTTCCACAAGGACGAAATGATCGCCCGCATCCACGCGGTGGTCCGTGGCTATGTGCTGCGCGACCCGAACGAGCAGGTTAACGCCGCCTGAggtaccacgt
**pGB1962**	pXMCS5	pXMCS5-YFP-TacA	agacgaccatATGAGCAAGGGCGAGGAGCTGTTCACCGGGGTGGTGCCCATCCTGGTCGAGCTGGACGGCGACGTAAACGGCCACAAGTTCAGCGTGTCCGGCGAGGGCGAGGGCGATGCCACCTACGGCAAGCTGACCCTGAAGTTCATCTGCACCACCGGCAAGCTGCCCGTGCCCTGGCCCACCCTCGTGACCACCTTCGGCTACGGCCTGCAGTGCTTCGCCCGCTACCCCGACCACATGAAGCAGCACGACTTCTTCAAGTCCGCCATGCCCGAAGGCTACGTCCAGGAGCGCACCATCTTCTTCAAGGACGACGGCAACTACAAGACCCGCGCCGAGGTGAAGTTCGAGGGCGACACCCTGGTGAACCGCATCGAGCTGAAGGGCATCGACTTCAAGGAGGACGGCAACATCCTGGGGCACAAGCTGGAGTACAACTACAACAGCCACAACGTCTATATCATGGCCGACAAGCAGAAGAACGGCATCAAGGTGAACTTCAAGATCCGCCACAACATCGAGGACGGCAGCGTGCAGCTCGCCGACCACTACCAGCAGAACACCCCCATCGGCGACGGCCCCGTGCTGCTGCCCGACAACCACTACCTGAGCTACCAGTCCGCCCTGAGCAAAGACCCCAACGAGAAGCGCGATCACATGGTCCTGCTGGAGTTCGTGACCGCCGCCGGGATCACTCTCGGCATGGACGAGCTGTACAAGGGAAGTAGCGGTGCGACCAAAACGGTCCTTGTCGTCGATGACGACCCGACACAACGTCGGTTGATTCAAGCGGTGCTCGAGCGCGACGGGTTCGCGGTCTCGCACGCGGAAGGCGGCGACGCGGCGATCGCGCACCTGACGTCCGGCGCGCCCGCCGATGTCATCCTGCTGGATCTCGTCATGCCTGGCCTCAACGGTCAGGACGCCCTGAAGGAAATGCGCGCCCGCGGCTTCAACCAGCCCGTGATCGTGCTGACGGCCAGCGGTGGCGTCGACACCGTGGTCAAGGCCATGCAGGCCGGCGCCTGCGACTTCTTCATCAAGCCCGCCTCGCCCGAGCGGATCACCGTGTCGATCCGCAACGCCCTGTCGATGGGCGACCTCAAGGGCGAGGTCGAGCGGCTGACCAAGCGCGCGGGCGGCAAGACCACCTTCGCGGACCTGATCGGCGCCTCGCCGGTCATGACCATGGTCAAGCGCATGGGCGAGCGGGCCGCCAAGAGCGGTATCCCGGTGCTGATCACCGGCGAAAGCGGCGTCGGCAAGGAGCTGATCGCCCGCGCCGTCCACGGCTCTTCCGACCGCGCCGGCAAGCCGTTTGTCGCGGTCAACTGCGGCGCGATCCCCGAGAACCTCGTCGAGTCGATCCTGTTCGGCCACGAGAAGGGCTCGTTCACCGGCGCCACCGACAAGCATCTGGGCAAGTTCAAGGAGGCCGACGGCGGCACCCTGTTCCTCGATGAGGTCGGCGAGCTGCCGCTCGACATGCAGGTCAAGCTGCTGCGCGCCCTGCAGGAGGGCGAGATCGACCCGATCGGCTCCAAGCGCTCGATCAAGGTCGATGTCCGGATCGTGTCGGCGACCAATCGCGATCTGCAGCAGGCCGTTTCGGGCGGCCGGTTCCGCGAAGACCTGTTTTATCGCCTGAACGTGTTCCCGATCGAGGCGCCGTCCCTGCGCGAGCGTCGCGAGGACATCCCGGCCCTCGTCGAGGCGTTCATCCGCCGGTTCAACGTCGAAGAAGGCAAGCGCGTGATCGGCGCCTCGCCCGAGACGATGCAACTGCTGACCAGCTTCGACTGGCCCGGCAATGTGCGCCAACTGGAAAACACCGTCTATCGCGCCATCGTGCTGGCCGATGCGCCCTATCTGCAGCCGTTCGACTTCCCGGCGATCTCGGGCCTGGCCGCGCCGATCGAGGCCGTATCGATCTCGCCCTCGCCGCCGCCTGCAGCCCTGTTGCAAGCCACGCATGCGGCGATGGCCGCCGCCGTCGCAGAGGCGCCTGTGCGCATCCTCGACGATCGCGGTCACCTGCGGACCCTGGAGGAGATCGAGCGCGACCTCATCCAGCATGCGATCGACGTCTATGCCGGCCACATGAGCGAGGTCGCGCGGCGTTTGGGCATCGGTCGCTCGACCCTCTATCGCAAGGTTCGCGAGCAGGGCATCGAAGTCGACATGAAGGAAGCGGGCTGAggtaccacgt

**pGB1963**	pXMCS5	pXMCS5-YFP-PdeA	agacgaccatATGAGCAAGGGCGAGGAGCTGTTCACCGGGGTGGTGCCCATCCTGGTCGAGCTGGA<br>CGGCGACGTAAACGGCCACAAGTTCAGCGTGTCCGGCGAGGGCGAGGGCGATGCCACCTACGGC<br>AAGCTGACCCTGAAGTTCATCTGCACCACCGGCAAGCTGCCCGTGCCCTGGCCCACCCTCGTGAC<br>CACCTTCGGCTACGGCCTGCAGTGCTTCGCCCGCTACCCCGACCACATGAAGCAGCACGACTTCTT<br>CAAGTCCGCCATGCCCGAAGGCTACGTCCAGGAGCGCACCATCTTCTTCAAGGACGACGGCAACT<br>ACAAGACCCGCGCCGAGGTGAAGTTCGAGGGCGACACCCTGGTGAACCGCATCGAGCTGAAGG<br>GCATCGACTTCAAGGAGGACGGCAACATCCTGGGGCACAAGCTGGAGTACAACTACAACAGCCA<br>CAACGTCTATATCATGGCCGACAAGCAGAAGAACGGCATCAAGGTGAACTTCAAGATCCGCCACA<br>ACATCGAGGACGGCAGCGTGCAGCTCGCCGACCACTACCAGCAGAACACCCCCATCGGCGACGG<br>CCCCGTGCTGCTGCCCGACAACCACTACCTGAGCTACCAGTCCGCCCTGAGCAAAGACCCCAACG<br>AGAAGCGCGATCACATGGTCCTGCTGGAGTTCGTGACCGCCGCCGGGATCACTCTCGGCATGGAC<br>GAGCTGTACAAGGGAAGTAGCGGTGCGTCTTTTCGGACAGGCGAACGACGAATCTGGGACGCGA<br>CGGCCACGCTTGAGGCGCTGGGCGCGGCGGACGTCGCCCTGTGGATCTGGGAGCCCGAAACCG<br>ACAGGTTGCGTCTGAACGGCGCGGCGCGCGCCTTGGGCCTTGGGCCGCTGGCGCCTGAATGCTC<br>GTCGGCCGCCTTCCGCGCCCTGGCCCTGCCGCAGGATCGCGCCCAGGCCGAAGAGGTCCTGAAG<br>CCGCGTGAACCGGGCAGCGAAGTCGTCGCCCGTTTCCGCGTGCGCGGCGGCGAGACCTGCCTCT<br>GGCGCGGCGTCTGGCTGGAAGAGGGCGTGCGCGCCGCCGGCGTCGTGGCGCCCGAAACGAAAT<br>TCTCCGCGTCCGAGCTTTGCGACCTGACCGGACTTCTGGACCGTCGCAGCTTCCTCGCCCGCGCCC<br>GCGAGCGCCTGGCGCAGGAGGGGACCCACCAGTTGGTCGTCGCCGACCTCGACCGACTGCGTCG<br>CCTGAACGAGGCGCTGGGTCACGAGCGCGCGGACCTAGTCCTGGCGGCGCTGGGCTCGCGCCTG<br>GCGGCGGCGTTCCCGGCCCAGTCGATCCTGGGCCGGATCGGCGAGGACGAGTTCGCCGTTCTCT<br>GCCAGCCGCTCGGCTACGAACCCTCCGATGTGCTGCGCAGCGCGCTGGAGCAGCCGCTGCGCGT<br>GGCCGGCTTTGATATTCACCCGACCCTGTCGATCGGCGCGGTCTCGGCCGAAGGCGGCCTGGACG<br>CGCCGGACGCCGCCGAACTGCTGCGCCGCGCCGAACTGGCCGTCGAGGCCGCCGCCGCCGCCG<br>GCCGAGGCGGCGCGGCCGCCTATGGCCGGGCGATGGAGACCGACGGCCTGTCGCGCCTGGCGC<br>TGGAGGCTGATCTGCGCGGCGCCATTGGTCGGGGCGAGATCACGCCCTACTTCCAGCCGATCGTG<br>CGGCTGTCGACCGGCGCCCTGTCCGGCTTCGAGGCCCTGGCCCGCTGGATCCATCCGCGCCGGG<br>GCATGCTGCCGCCGGACGAGTTCCTGCCGCTGATCGAAGAGATGGGGCTGATGAGCGAGCTCGG<br>CGCGCACATGATGCACGCCGCCGCCCAGCAGCTGTCGACCTGGCGCGCCGCTCACCCGGCGATGG<br>GGAACCTGACTGTCAGCGTCAACCTGTCGACCGGCGAGATCGACCGGCCCGGCCTGGTCGCCGA<br>CGTGGCCGAGACCCTGCGGGTCAATCGCCTGCCGCGCGGCGCCCTGAAGCTGGAAGTCACCGAA<br>AGCGACATCATGCGCGATCCCGAACGGGCCGCCGTGATCCTGAAGACGCTGCGCGACGCGGGCG<br>CAGGGCTTGCGCTGGACGACTTCGGCACCGGCTTCTCGTCGCTGTCGTACCTGACGCGCCTGCCG<br>TTCGACACGCTGAAGATCGACCGCTACTTCGTCCGCACCATGGGCAATAACGCCGGCTCGGCCAA<br>GATCGTCCGCTCGGTGGTCAAGCTGGGCCAGGATCTGGATCTGGAAGTCGTCGCCGAAGGGGTC<br>GAGAACGCCGAGATGGCGCATGCGCTGCAATCGCTGGGCTGTGACTATGGCCAAGGCTTTGGCTA<br>TGCGCCGGCCCTGTCGCCGCAGGAGGCCGAGGTCTATCTGAACGAGGCCTATGTCGACGGCGCC<br>GCGCCGGTGAAGGCGCGGGGTTAAggtaccacgt
**pGB1964**	pXMCS5	pXMCS5-HA-CtrA RD+15	agacgaccatATGTACCCATACGATGTTCCAGATTACGCAGGAAGTCGCGTACTGTTGATCGAGGATG<br>ACAGCGCGACGGCGCAGACCATCGAACTGATGCTGAAGTCTGAAGGCTTCAACGTCTATACGACG<br>GATCTGGGTGAAGAAGGCGTCGATCTGGGCAAGATCTACGACTACGATCTTATCCTGCTCGACCTC<br>AATCTTCCGGACATGAGCGGCATCGATGTTCTGCGCACCCTGCGGGTCGCGAAGATCAACACGCC<br>CATCATGATCCTGTCGGGCTCGTCGGAAATCGACACCAAGGTCAAGACCTTCGCCGGCGGCGCCG<br>ACGACTACATGACCAAGCCGTTCCACAAGGACGAAATGATCGCCCGCATCCACGCGGTGGTCCGT<br>GGCTATGTGCTGCGCGACCCGAACGAGCAGGTTAACGCCGCCTGAggtaccacgt
**pGB1965**	pXMCS5	pXMCS5-HA-TacA	agacgaccatATGTACCCATACGATGTTCCAGATTACGCAGGAAGTACCAAAACGGTCCTTGTCGTCG<br>ATGACGACCCGACACAACGTCGGTTGATTCAAGCGGTGCTCGAGCGCGACGGGTTCGCGGTCTC<br>GCACGCGGAAGGCGGCGACGCGGCGATCGCGCACCTGACGTCCGGCGCGCCCGCCGATGTCATC<br>CTGCTGGATCTCGTCATGCCTGGCCTCAACGGTCAGGACGCCCTGAAGGAAATGCGCGCCCGCGG<br>CTTCAACCAGCCCGTGATCGTGCTGACGGCCAGCGGTGGCGTCGACACCGTGGTCAAGGCCATGC<br>AGGCCGGCGCCTGCGACTTCTTCATCAAGCCCGCCTCGCCCGAGCGGATCACCGTGTCGATCCGC<br>AACGCCCTGTCGATGGGCGACCTCAAGGGCGAGGTCGAGCGGCTGACCAAGCGCGCGGGCGGC<br>AAGACCACCTTCGCGGACCTGATCGGCGCCTCGCCGGTCATGACCATGGTCAAGCGCATGGGCGA<br>GCGGGCCGCCAAGAGCGGTATCCCGGTGCTGATCACCGGCGAAAGCGGCGTCGGCAAGGAGCT<br>GATCGCCCGCGCCGTCCACGGCTCTTCCGACCGCGCCGGCAAGCCGTTTGTCGCGGTCAACTGCG

			GCGCGATCCCCGAGAACCTCGTCGAGTCGATCCTGTTCGGCCACGAGAAGGGCTCGTTCACCGGC<br>GCCACCGACAAGCATCTGGGCAAGTTCAAGGAGGCCGACGGCGGCACCCTGTTCCTCGATGAGG<br>TCGGCGAGCTGCCGCTCGACATGCAGGTCAAGCTGCTGCGCGCCCTGCAGGAGGGCGAGATCGA<br>CCCGATCGGCTCCAAGCGCTCGATCAAGGTCGATGTCCGGATCGTGTCGGCGACCAATCGCGATCT<br>GCAGCAGGCCGTTTCGGGCGGCCGGTTCCGCGAAGACCTGTTTTATCGCCTGAACGTGTTCCCGA<br>TCGAGGCGCCGTCCCTGCGCGAGCGTCGCGAGGACATCCCGGCCCTCGTCGAGGCGTTCATCCGC<br>CGGTTCAACGTCGAAGAAGGCAAGCGCGTGATCGGCGCCTCGCCCGAGACGATGCAACTGCTGA<br>CCAGCTTCGACTGGCCCGGCAATGTGCGCCAACTGGAAAACACCGTCTATCGCGCCATCGTGCTG<br>GCCGATGCGCCCTATCTGCAGCCGTTCGACTTCCCGGCGATCTCGGGCCTGGCCGCGCCGATCGA<br>GGCCGTATCGATCTCGCCCTCGCCGCCGCCTGCAGCCCTGTTGCAAGCCACGCATGCGGCGATGG<br>CCGCCGCCGTCGCAGAGGCGCCTGTGCGCATCCTCGACGATCGCGGTCACCTGCGGACCCTGGAG<br>GAGATCGAGCGCGACCTCATCCAGCATGCGATCGACGTCTATGCCGGCCACATGAGCGAGGTCGC<br>GCGGCGTTTGGGCATCGGTCGCTCGACCCTCTATCGCAAGGTTCGCGAGCAGGGCATCGAAGTCG<br>ACATGAAGGAAGCGGGCTGAggtaccacgt
**pGB1966**	pXMCS5	pXMCS5-HA-PdeA	agacgaccatATGTACCCATACGATGTTCCAGATTACGCAGGAAGTTCTTTTCGGACAGGCGAACGAC<br>GAATCTGGGACGCGACGGCCACGCTTGAGGCGCTGGGCGCGGCGGACGTCGCCCTGTGGATCTG<br>GGAGCCCGAAACCGACAGGTTGCGTCTGAACGGCGCGGCGCGCGCCTTGGGCCTTGGGCCGCT<br>GGCGCCTGAATGCTCGTCGGCCGCCTTCCGCGCCCTGGCCCTGCCGCAGGATCGCGCCCAGGCCG<br>AAGAGGTCCTGAAGCCGCGTGAACCGGGCAGCGAAGTCGTCGCCCGTTTCCGCGTGCGCGGCG<br>GCGAGACCTGCCTCTGGCGCGGCGTCTGGCTGGAAGAGGGCGTGCGCGCCGCCGGCGTCGTGG<br>CGCCCGAAACGAAATTCTCCGCGTCCGAGCTTTGCGACCTGACCGGACTTCTGGACCGTCGCAGC<br>TTCCTCGCCCGCGCCCGCGAGCGCCTGGCGCAGGAGGGGACCCACCAGTTGGTCGTCGCCGACC<br>TCGACCGACTGCGTCGCCTGAACGAGGCGCTGGGTCACGAGCGCGCGGACCTAGTCCTGGCGGC<br>GCTGGGCTCGCGCCTGGCGGCGGCGTTCCCGGCCCAGTCGATCCTGGGCCGGATCGGCGAGGAC<br>GAGTTCGCCGTTCTCTGCCAGCCGCTCGGCTACGAACCCTCCGATGTGCTGCGCAGCGCGCTGGA<br>GCAGCCGCTGCGCGTGGCCGGCTTTGATATTCACCCGACCCTGTCGATCGGCGCGGTCTCGGCCG<br>AAGGCGGCCTGGACGCGCCGGACGCCGCCGAACTGCTGCGCCGCGCCGAACTGGCCGTCGAGG<br>CCGCCGCCGCCGCCGGCCGAGGCGGCGCGGCCGCCTATGGCCGGGCGATGGAGACCGACGGCC<br>TGTCGCGCCTGGCGCTGGAGGCTGATCTGCGCGGCGCCATTGGTCGGGGCGAGATCACGCCCTAC<br>TTCCAGCCGATCGTGCGGCTGTCGACCGGCGCCCTGTCCGGCTTCGAGGCCCTGGCCCGCTGGAT<br>CCATCCGCGCCGGGGCATGCTGCCGCCGGACGAGTTCCTGCCGCTGATCGAAGAGATGGGGCTG<br>ATGAGCGAGCTCGGCGCGCACATGATGCACGCCGCCGCCCAGCAGCTGTCGACCTGGCGCGCCG<br>CTCACCCGGCGATGGGGAACCTGACTGTCAGCGTCAACCTGTCGACCGGCGAGATCGACCGGCCC<br>GGCCTGGTCGCCGACGTGGCCGAGACCCTGCGGGTCAATCGCCTGCCGCGCGGCGCCCTGAAGC<br>TGGAAGTCACCGAAAGCGACATCATGCGCGATCCCGAACGGGCCGCCGTGATCCTGAAGACGCTG<br>CGCGACGCGGGCGCAGGGCTTGCGCTGGACGACTTCGGCACCGGCTTCTCGTCGCTGTCGTACCT<br>GACGCGCCTGCCGTTCGACACGCTGAAGATCGACCGCTACTTCGTCCGCACCATGGGCAATAACG<br>CCGGCTCGGCCAAGATCGTCCGCTCGGTGGTCAAGCTGGGCCAGGATCTGGATCTGGAAGTCGTC<br>GCCGAAGGGGTCGAGAACGCCGAGATGGCGCATGCGCTGCAATCGCTGGGCTGTGACTATGGCC<br>AAGGCTTTGGCTATGCGCCGGCCCTGTCGCCGCAGGAGGCCGAGGTCTATCTGAACGAGGCCTAT<br>GTCGACGGCGCCGCGCCGGTGAAGGCGCGGGGTTAAggtaccacgt
**pGB1967**	pVMCS6 (NdeI, KpnI)	pVMCS6-ClpX-GFP	ggaaacgcatATGACGAAAGCCGCGAGCGGCGACACGAAAAGCACCCTGTACTGCTCTTTCTGCGG<br>AAAGAGCCAACATGAGGTGCGCAAGCTCATCGCGGGACCGACGGTGTTCATTTGCGATGAATGCG<br>TCGAGCTCTGCATGGACATCATCCGCGAAGAGCACAAGATCGCCTTCGTGAAGTCTAAGGACGGC<br>GTCCCGACGCCGCGCGAAATCTGCGAAGTCCTGGATGATTACGTGATCGGTCAAGGTCACGCCAA<br>GAAGGTCCTCGCGGTCGCAGTGCACAATCACTACAAGCGGCTGAACCACGCTTCGAAGAATAACG<br>ACGTCGAACTGGCCAAGTCGAACATCCTGCTGGTCGGTCCGACCGGTACGGGTAAGACCCTGCTG<br>GCGCAGACGCTGGCCCGAATCATCGACGTTCCGTTCACGATGGCGGACGCCACGACGCTGACCGA<br>AGCCGGTTACGTCGGCGAAGACGTCGAGAACATCGTGCTGAAGCTGCTGCAGGCCGCCGACTAC<br>AACGTCGAGCGCGCCCAGCGCGGCATCGTCTACATCGACGAAATCGACAAGATCAGCCGCAAGTC<br>CGACAACCCGTCGATCACTCGCGACGTGTCGGGCGAGGGCGTGCAGCAGGCTCTGCTGAAGATC<br>ATGGAAGGCACGGTCGCCTCCGTGCCGCCGCAGGGCGGGCGCAAGCATCCTCAGCAGGAGTTCC<br>TGCAGGTCGACACGACGAACATCCTGTTCATCTGTGGCGGCGCCTTCGCTGGCCTGGAGAAGATC<br>ATCTCGGCGCGCGGCGCGGCCAAGTCGATCGGCTTCGGCGCCAAGGTGACCGATCCCGAAGAGC<br>GCCGGACGGGCGAGATCCTTCGGAACGTCGAGCCCGACGACCTGCAGCGTTTCGGCCTGATCCC

			GGAGTTCATCGGCCGTCTGCCGGTGGTCGCCACGCTGGAGGATCTGGACGAGGCCGCCCTGGTC<br>AAGATCCTGACCGAGCCGAAGAACGCCTTCGTCAAGCAGTATCAGCGCCTGTTCGAGATGGAGAA<br>CATCGGCCTGACCTTCACCGAAGACGCTCTGCATCAGGTGGCCAAGAAGGCTATCGCGCGCAAGA<br>CCGGCGCGCGCGGCCTGCGCTCGATCATGGAAGGCATCCTGCTGGAGACCATGTTCGAACTGCCG<br>ACCTACGAGGGCGTCGAGGAAGTGGTGGTCAACGCCGAGGTCGTCGAAGGCCGGGCTCAGCCG<br>CTGCTGATCTATGCCGAGAAAAAGGGTGGGGCGGCATCGGCCGGAAGTAGTAAAGGTGAAGAAC<br>TGTTCACCGGTGTTGTTCCGATCCTGGTTGAACTGGATGGTGATGTTAACGGCCACAAATTCTCTGT<br>TCGTGGTGAAGGTGAAGGTGATGCAACCAACGGTAAACTGACCCTGAAATTCATCTGCACTACCG<br>GTAAACTGCCGGTTCCATGGCCGACTCTGGTGACTACCCTGACCTATGGTGTTCAGTGTTTTTCTCG<br>TTACCCGGATCACATGAAGCAGCATGATTTCTTCAAATCTGCAATGCCGGAAGGTTATGTACAGGA<br>GCGCACCATTTCTTTCAAAGACGATGGCACCTACAAAACCCGTGCAGAGGTTAAATTTGAAGGTG<br>ATACTCTGGTGAACCGTATTGAACTGAAAGGCATTGATTTCAAAGAGGACGGCAACATCCTGGGC<br>CACAAACTGGAATATAACTTCAACTCCCATAACGTTTACATCACCGCAGACAAACAGAAGAACGGT<br>ATCAAAGCTAACTTCAAAATTCGCCATAACGTTGAAGACGGTAGCGTACAGCTGGCGGACCACTAC<br>CAGCAGAACACTCCGATCGGTGATGGTCCGGTTCTGCTGCCGGATAACCACTACCTGTCCACCCAG<br>TCTAAACTGTCCAAAGACCCGAACGAAAAGCGCGACCACATGGTGCTGCTGGAGTTCGTTACTGC<br>AGCAGGTATCACGCACGGCATGGATGAACTCTACAAATAGggtaccacgt
**pGB1968**	pVMCS6	pVMCS6-RcdA-GFP	ggaaacgcatATGACCGAAGTGAACGCGTTCGCGGACACGCCTTGGCGCGCTGGAGTGATCCAGGA<br>TTTCGCGCGATCGGAACTGTTCGACCGGACGTTCGAGGAAGGCATGCAACTGGTAGAAGAGACC<br>GCCGCCTATCTCGACGGGGCCGGACGCCATGACAGCAAGGTCCTCTCTCGCAACGCCGCCCTGGG<br>CTACGCCACCGAAAGCATGCGCCTGACCACGCGCCTGATGCAGGTCGCCTCCTGGCTTTTGGTGC<br>AGCGCGCCGTACGTGAAGGCGAGATGCCGCCGGAAGCCGCCTGCGCTGAAGCCTATCGCCTGGC<br>CGAAGAGGCCCCGGCCGATGGTCCGGCCGTCGAGGAACTGCCGTTTGGCCTGATGAACCTGCTG<br>CAGCGCTCCGAGCGCCTGTACGAGCGCGTCCGCCACCTGGACCGCCGCATGTATGTCGAGTCGCC<br>GAACGAAGAAGCGCCGCGTCCGGTTCAGAACCAGCTCGATCGCTTGACGGCGGCGTTCGGAGGC<br>AGCGGCGGCGGCGGCAGTAAAGGTGAAGAACTGTTCACCGGTGTTGTTCCGATCCTGGTTGAAC<br>TGGATGGTGATGTTAACGGCCACAAATTCTCTGTTCGTGGTGAAGGTGAAGGTGATGCAACCAAC<br>GGTAAACTGACCCTGAAATTCATCTGCACTACCGGTAAACTGCCGGTTCCATGGCCGACTCTGGTG<br>ACTACCCTGACCTATGGTGTTCAGTGTTTTTCTCGTTACCCGGATCACATGAAGCAGCATGATTTCTT<br>CAAATCTGCAATGCCGGAAGGTTATGTACAGGAGCGCACCATTTCTTTCAAAGACGATGGCACCTA<br>CAAAACCCGTGCAGAGGTTAAATTTGAAGGTGATACTCTGGTGAACCGTATTGAACTGAAAGGCA<br>TTGATTTCAAAGAGGACGGCAACATCCTGGGCCACAAACTGGAATATAACTTCAACTCCCATAACG<br>TTTACATCACCGCAGACAAACAGAAGAACGGTATCAAAGCTAACTTCAAAATTCGCCATAACGTTG<br>AAGACGGTAGCGTACAGCTGGCGGACCACTACCAGCAGAACACTCCGATCGGTGATGGTCCGGTT<br>CTGCTGCCGGATAACCACTACCTGTCCACCCAGTCTAAACTGTCCAAAGACCCGAACGAAAAGCGC<br>GACCACATGGTGCTGCTGGAGTTCGTTACTGCAGCAGGTATCACGCACGGCATGGATGAACTCTAC<br>AAATAGggtaccacgt
**pGB1969**	pACYC-duet (NcoI, XhoI)	pACYC-CpdR-TEV-GFP-6XHis	gagatataccATGGCAAGAATTCTCCTCGCTGAAGATGATGATTCTCTGAGAGGATTTCTGGCTAGAG<br>CTCTGGAAAGAGCTGGATTTGAAGTCCAGGCTTGCGCTGATGGAGAAGAAGCTGTCCAGCACCT<br>GGATCATCCTTGGGATCTGCTGCTGACAGATATTGTCATGCCTGGAATGGATGGAATTGAAGTGGC<br>TAGACAGGCTGCTGCTAGAGATCCGAGTCTGAGAATTATGTTTATTACAGGATTTGCTGCTGTGGCT<br>CTCTCGGCTCAGGATAGAGCTCCTGCTGGAGCTAAGGTGCTGAGTAAGCCTGTGCACCTGAGAGA<br>TCTCGTCGCTGAAGTCGAAAAGATGATGGCTGCTGAAAACCTGTATTTTCAGGGTAGCAGTAAAG<br>GTGAAGAACTGTTCACCGGTGTTGTTCCGATCCTGGTTGAACTGGATGGTGATGTTAACGGCCACA<br>AATTCTCTGTTCGTGGTGAAGGTGAAGGTGATGCAACCAACGGTAAACTGACCCTGAAATTCATCT<br>GCACTACCGGTAAACTGCCGGTTCCATGGCCGACTCTGGTGACTACCCTGACCTATGGTGTTCAGT<br>GTTTTTCTCGTTACCCGGATCACATGAAGCAGCATGATTTCTTCAAATCTGCAATGCCGGAAGGTTA<br>TGTACAGGAGCGCACCATTTCTTTCAAAGACGATGGCACCTACAAAACCCGTGCAGAGGTTAAATT<br>TGAAGGTGATACTCTGGTGAACCGTATTGAACTGAAAGGCATTGATTTCAAAGAGGACGGCAACA<br>TCCTGGGCCACAAACTGGAATATAACTTCAACTCCCATAACGTTTACATCACCGCAGACAAACAGA<br>AGAACGGTATCAAAGCTAACTTCAAAATTCGCCATAACGTTGAAGACGGTAGCGTACAGCTGGCG<br>GACCACTACCAGCAGAACACTCCGATCGGTGATGGTCCGGTTCTGCTGCCGGATAACCACTACCTG<br>TCCACCCAGTCTaaaCTGTCCAAAGACCCGAACGAAAAGCGCGACCACATGGTGCTGCTGGAGTTC<br>GTTACTGCAGCAGGTATCACGCACGGCATGGATGAACTCTACAAACACCATCACCATCACCATTAGc<br>tcgagtctg

**pGB1970**	pACYC-duet	pACYC-CpdR$_{D51A}$-TEV-GFP-6XHis	gagatataccATGGCAAGAATTCTCCTCGCTGAAGATGATGATTCTCTGAGAGGATTTCTGGCTAGAGCTCTGGAAAGAGCTGGATTTGAAGTCCAGGCTTGCGCTGATGGAGAAGAAGCTGTCCAGCACCTGGATCATCCTTGGGATCTGCTGCTGACAGCCATTGTCATGCCTGGAATGGATGGAATTGAAGTGGCTAGACAGGCTGCTGCTAGAGATCCGAGTCTGAGAATTATGTTTATTACAGGATTTGCTGCTGTGGCTCTCTCGGCTCAGGATAGAGCTCCTGCTGGAGCTAAGGTGCTGAGTAAGCCTGTGCACCTGAGAGATCTCGTCGCTGAAGTCGAAAAGATGATGGCTGCTGAAAACCTGTATTTTCAGGGTAGCAGTAAAGGTGAAGAACTGTTCACCGGTGTTGTTCCGATCCTGGTTGAACTGGATGGTGATGTTAACGGCCACAAATTCTCTGTTCGTGGTGAAGGTGAAGGTGATGCAACCAACGGTAAACTGACCCTGAAATTCATCTGCACTACCGGTAAACTGCCGGTTCCATGGCCGACTCTGGTGACTACCCTGACCTATGGTGTTCAGTGTTTTTCTCGTTACCCGGATCACATGAAGCAGCATGATTTCTTCAAATCTGCAATGCCGGAAGGTTATGTACAGGAGCGCACCATTTCTTTCAAAGACGATGGCACCTACAAAACCCGTGCAGAGGTTAAATTTGAAGGTGATACTCTGGTGAACCGTATTGAACTGAAAGGCATTGATTTCAAAGAGGACGGCAACATCCTGGGCCACAAACTGGAATATAACTTCAACTCCCATAACGTTTACATCACCGCAGACAAACAGAAGAACGGTATCAAAGCTAACTTCAAAATTCGCCATAACGTTGAAGACGGTAGCGTACAGCTGGCGGACCACTACCAGCAGAACACTCCGATCGGTGATGGTCCGGTTCTGCTGCCGGATAACCACTACCTGTCCACCCAGTCTaaaCTGTCCAAAGACCCGAACGAAAAGCGCGACCACATGGTGCTGCTGGAGTTCGTTACTGCAGCAGGTATCACGCACGGCATGGATGAACTCTACAAACACCATCACCATCACCATTAGctcgagtctg
**pGB2010**		pBXMCS2-pCpdR CpdR	agacgaccatTCACGGTTCAGATCCACATAGGCGCGGGCGAAGCGGGCGCGCACCACGGCGGCCCCCAGGGCGGGCGCGCCGCCGATGATGCGGTCGACAAACGCGTCCTCGGACGCGCGCAGGGTCTCGAGCGGCAGGCGGACCGCGGCGACCATGTCCTCCGGATAGAGGTCGCCCGAGTGCGGCGAGGCGAACACCAAGGCGGTCGGCGGCGGCGCCCCGGCCGGGACCGCGCGCAGCACCTCGAACGCTGGCCCGCCAAAGGTCTCCAGCGGCGGGGACGAAAGCTCTTCGCTCGAAAGGGGCCGCCACGCGCTCATGCCGCATCATCGAAGCGTCGCGGCGCGGGGTCAAATCTCAATCGCCGCTGACGAAACATCCCCAGCCGCGACGTTTAGGTTCATCCCCGATTTACGGACGGGGCGATAAGGTGGATCCTCTATCGACGATCTTAATCGGACACGTGACCCCatgGCCCGCATCCTCCTCGCCGAAGACGATGATTCCCTGCGCGGCTTCCTGGCCCGCGCGCTGGAACGCGCCGGCTTCGAAGTCCAGGCCTGCGCCGACGGCGAAGAGGCCGTCCAGCACCTGGACCATCCCTGGGACCTGCTGCTGACCGACATCGTCATGCCCGGCATGGACGGCATCGAGGTGGCCCGCCAGGCCGCCGCCCGCGACCCGTCCCTGCGCATCATGTTCATCACCGGCTTCGCCGCCGTGGCCCTCTCGGCCCAGGACCGCGCGCCCGCCGGCGCCAAGGTGCTGTCCAAGCCCGTGCACCTGCGCGACCTCGTCGCCGAGGTCGAAAAGATGATGGCGGCCtgagaattcctgc
**pGB2011**		pBXMCS2-pCpdR CpdR$_{D51A}$	agacgaccatTCACGGTTCAGATCCACATAGGCGCGGGCGAAGCGGGCGCGCACCACGGCGGCCCCCAGGGCGGGCGCGCCGCCGATGATGCGGTCGACAAACGCGTCCTCGGACGCGCGCAGGGTCTCGAGCGGCAGGCGGACCGCGGCGACCATGTCCTCCGGATAGAGGTCGCCCGAGTGCGGCGAGGCGAACACCAAGGCGGTCGGCGGCGGCGCCCCGGCCGGGACCGCGCGCAGCACCTCGAACGCTGGCCCGCCAAAGGTCTCCAGCGGCGGGGACGAAAGCTCTTCGCTCGAAAGGGGCCGCCACGCGCTCATGCCGCATCATCGAAGCGTCGCGGCGCGGGGTCAAATCTCAATCGCCGCTGACGAAACATCCCCAGCCGCGACGTTTAGGTTCATCCCCGATTTACGGACGGGGCGATAAGGTGGATCCTCTATCGACGATCTTAATCGGACACGTGACCCCatgGCCCGCATCCTCCTCGCCGAAGACGATGATTCCCTGCGCGGCTTCCTGGCCCGCGCGCTGGAACGCGCCGGCTTCGAAGTCCAGGCCTGCGCCGACGGCGAAGAGGCCGTCCAGCACCTGGACCATCCCTGGGACCTGCTGCTGACCGCCATCGTCATGCCCGGCATGGACGGCATCGAGGTGGCCCGCCAGGCCGCCGCCCGCGACCCGTCCCTGCGCATCATGTTCATCACCGGCTTCGCCGCCGTGGCCCTCTCGGCCCAGGACCGCGCGCCCGCCGGCGCCAAGGTGCTGTCCAAGCCCGTGCACCTGCGCGACCTCGTCGCCGAGGTCGAAAAGATGATGGCGGCCtgagaattcctgc

**pGB2012**		pBXMCS2-pXyl-CckA H+ pCpdR CpdR	agacgaccatATGGCCGACTTGCAGCTCCAGGACAAGGTTTCGACCGGCGCCCCGCGTCGGCGGTTT<br>GATCCATGGCTGGTCGGCGCGGCGGTGTTTTTCGTGGCTGCGGCGGCCCTCTCGGCGGCGCCGG<br>CGCTCAAGGCCGGACCGACCACCCTGGCCGGCCTGCTGCTGCTTCTGGGCGTCGCGGGCGTGGC<br>TGTGTTGGGCCTTGTCGCCATTCGCGGCTCAGCGCTTTCCGGCGGCGACGCCGACCAGGCTGAG<br>GGGTTCATCGAGGCGCTGGCCGAGCCGGCCGCCCTGGCCGCCGCCGACGGTCGCGTCCTGGCCG<br>CCAACGGGCCCTGGCGCGAAGTCATGGGCGAACAGCGCCGCCTGCCCAAGGGCGTGGCGGGCT<br>CCAGCCTGTTTGCGGCCCTGGTCCAGGCGCGCCAGGGGCAGATGGCCGAGGGCATGCTGAGCGC<br>TGGAGGAACCGACTATACCGCCAAGGTCTCGCGCCTTGCGGGCGGACGGCTGATGATCCGGCTTG<br>CGCCCATCGTTGTCGCTGAGCCGGTTGTGGAAGACGCGTCGCCGGCCCCGGTCGCCGAGCGCGC<br>CGCCCCGCCACCCAGCTCGCTGGACGCCTTCGCCGGAGCCTCGCCGTTCGGCGCGGCCCTGCTGG<br>AAGGCCTGGAGCCGTTCACCTCGCGGGTGCTGGAGACCAATCCGGCGCTGACCACGATGACCGG<br>CGCCAAGGCCGGTGTGCTGTTCGGCGATCTGATCGACGCCGCCTCGCGCGCCGAGGCCGAGACG<br>CGCCTGAACGAAGGCCGCGCCGGTCCCTACGAGGTGCGGTTGGCGCGCGATCCGTCGCGCATCG<br>CTCACCTTTACCTCTATCGCGCCGAAGGCCGCCTGGTGGCCTACATGATCGACGTGTCCGAGCAGA<br>AGCAGATCGAGCTGCAGCTGTCCCAGGCCCAGAAGATGCAGGCCATCGGCCAGCTGGCCGGCGG<br>CGTCGCGCACGACTTCAACAACCTCTTGACCGCCATCCAGCTGCGTCTGGACGAGTTGCTGCATCG<br>CCATCCCGTCGGTGATCCGTCGTACGAGGGTCTCAACGAGATCCGTCAGACGGGCGTGCGCGCCG<br>CCGACCTCGTGCGCAAGCTCTTGGCTTTCTCGCGCAAGCAGACCGTGCAGCGCGAGGTGCTGGAT<br>CTGGGCGAGCTGATCAGCGAGTTCGAGGTCTTGCTGCGCCGCCTGCTGCGCGAAGACGTCAAGC<br>TGATCACCGACTATGGCCGCGACCTGCCGCAGGTGCGCGCCGACAAGAGCCAGCTCGAGACGGC<br>GGTCATGAACCTGGCCGTCAACGCCCGCGACGCCGTGCGCGCGGCCAAGGGCGGCGGCGTCGT<br>GCGCATCCGCACCGCGCGCCTGACCCGCGACGAGGCGATCCAGCTGGGTTTCCCGGCCGCCGAC<br>GGCGACACGGCCTTCATTGAGGTCAGTGACGATGGTCCGGGCATTCCGCCCGACGTCATGGGCAA<br>GATCTTCGACCCGTTCTTCACCACCAAGCCGGTGGGCGAGGGTACGGGCCTAGGCCTAGCCACGG<br>TCTATGGCATCGTTAAGCAGAGCGACGGCTGGATTCACGTCCACAGCCGTCCGAACGAAGGCGCG<br>GCCTTCCGCATCTTCCTGCCGGTCTATGAAGCGCCCGCCGGCGCGGTCGCCGTCCAGGCCGTCGC<br>CGAGCCCGCCAAGCCGCGCGCCGCTCGCGACCTGTCGGGCGCCGGCCGCATCCTGTTCGTCGAG<br>GACGAGGACGCCGTGCGCAGCGTCGCCGCCCGCCTGCTGCGCGCCCGTGGCTACGAGGTGCTTG<br>AGGCGGCCGACGGCGAAGAGGCCCTGATCATCGCCGAGGAAAACGCCGGCACGATCGACCTCTT<br>GATCAGCGACGTGATCATGCCCGGCATCGACGGCCCGACCTTGCTGAAGAAGGCGCGTGGCTATC<br>TGGGGACCGCGCCGGTGATGTTCATCTCCGGCTATGCCGAGGCCGAGTTCAGCGACCTCTTGGAA<br>GGCGAGACGGGCGTGACCTTCCTCCCCAAGCCGATCGACATCAAGACCCTGGCCGAGCGCGTCA<br>AGCAGCAGCTGCAAGCAGCATAGtctgtgctattctaacgaatcttgattcgTCACGGTTCAGATCCACATAG<br>GCGCGGGCGAAGCGGGCGCGCACCACGGCGGCCCCCAGGGCGGGCGCGCCGCCGATGATGCG<br>GTCGACAAACGCGTCCTCGGACGCGCGCAGGGTCTCGAGCGGCAGGCGGACCGCGGCGACCAT<br>GTCCTCCGGATAGAGGTCGCCCGAGTGCGGCGAGGCGAACACCAAGGCGGTCGGCGGCGGCGC<br>CCCGGCCGGGACCGCGCGCAGCACCTCGAACGCTGGCCCGCCAAAGGTCTCCAGCGGCGGGGA<br>CGAAAGCTCTTCGCTCGAAAGGGGCCGCCACGCGCTCATGCCGCATCATCGAAGCGTCGCGGCGC<br>GGGGTCAAATCTCAATCGCCGCTGACGAAACATCCCCAGCCGCGACGTTTAGGTTCATCCCCGATT<br>TACGGACGGGGCGATAAGGTGGATCCTCTATCGACGATCTTAATCGGACACGTGACCCCatgGCCC<br>GCATCCTCCTCGCCGAAGACGATGATTCCCTGCGCGGCTTCCTGGCCCGCGCGCTGGAACGCGCC<br>GGCTTCGAAGTCCAGGCCTGCGCCGACGGCGAAGAGGCCGTCCAGCACCTGGACCATCCCTGGG<br>ACCTGCTGCTGACCGACATCGTCATGCCCGGCATGGACGGCATCGAGGTGGCCCGCCAGGCCGCC<br>GCCCGCGACCCGTCCCTGCGCATCATGTTCATCACCGGCTTCGCCGCCGTGGCCCTCTCGGCCCAG<br>GACCGCGCGCCCGCCGGCGCCAAGGTGCTGTCCAAGCCCGTGCACCTGCGCGACCTCGTCGCCG<br>AGGTCGAAAAGATGATGGCGGCCtgagaattcctgc

**pGB2013**		pBXMCS2-pXyl-CckA H+ pCpdR CpdR$_{D51A}$	agacgaccatATGGCCGACTTGCAGCTCCAGGACAAGGTTTCGACCGGCGCCCCGCGTCGGCGGTTT<br>GATCCATGGCTGGTCGGCGCGGCGGTGTTTTTCGTGGCTGCGGCGGCCCTCTCGGCGGCGCCGG<br>CGCTCAAGGCCGGACCGACCACCCTGGCCGGCCTGCTGCTGCTTCTGGGCGTCGCGGGCGTGGC<br>TGTGTTGGGCCTTGTCGCCATTCGCGGCTCAGCGCTTTCCGGCGGCGACGCCGACCAGGCTGAG<br>GGGTTCATCGAGGCGCTGGCCGAGCCGGCCGCCCTGGCCGCCGCCGACGGTCGCGTCCTGGCCG<br>CCAACGGGCCCTGGCGCGAAGTCATGGGCGAACAGCGCCGCCTGCCCAAGGGCGTGGCGGGCT<br>CCAGCCTGTTTGCGGCCCTGGTCCAGGCGCGCCAGGGGCAGATGGCCGAGGGCATGCTGAGCGC<br>TGGAGGAACCGACTATACCGCCAAGGTCTCGCGCCTTGCGGGCGGACGGCTGATGATCCGGCTTG<br>CGCCCATCGTTGTCGCTGAGCCGGTTGTGGAAGACGCGTCGCCGGCCCCGGTCGCCGAGCGCGC<br>CGCCCCGCCACCCAGCTCGCTGGACGCCTTCGCCGGAGCCTCGCCGTTCGGCGCGGCCCTGCTGG<br>AAGGCCTGGAGCCGTTCACCTCGCGGGTGCTGGAGACCAATCCGGCGCTGACCACGATGACCGG<br>CGCCAAGGCCGGTGTGCTGTTCGGCGATCTGATCGACGCCGCCTCGCGCGCCGAGGCCGAGACG<br>CGCCTGAACGAAGGCCGCGCCGGTCCCTACGAGGTGCGGTTGGCGCGCGATCCGTCGCGCATCG<br>CTCACCTTTACCTCTATCGCGCCGAAGGCCGCCTGGTGGCCTACATGATCGACGTGTCCGAGCAGA<br>AGCAGATCGAGCTGCAGCTGTCCCAGGCCCAGAAGATGCAGGCCATCGGCCAGCTGGCCGGCGG<br>CGTCGCGCACGACTTCAACAACCTCTTGACCGCCATCCAGCTGCGTCTGGACGAGTTGCTGCATCG<br>CCATCCCGTCGGTGATCCGTCGTACGAGGGTCTCAACGAGATCCGTCAGACGGGCGTGCGCGCCG<br>CCGACCTCGTGCGCAAGCTCTTGGCTTTCTCGCGCAAGCAGACCGTGCAGCGCGAGGTGCTGGAT<br>CTGGGCGAGCTGATCAGCGAGTTCGAGGTCTTGCTGCGCCGCCTGCTGCGCGAAGACGTCAAGC<br>TGATCACCGACTATGGCCGCGACCTGCCGCAGGTGCGCGCCGACAAGAGCCAGCTCGAGACGGC<br>GGTCATGAACCTGGCCGTCAACGCCCGCGACGCCGTGCGCGCGGCCAAGGGCGGCGGCGTCGT<br>GCGCATCCGCACCGCGCGCCTGACCCGCGACGAGGCGATCCAGCTGGGTTTCCCGGCCGCCGAC<br>GGCGACACGGCCTTCATTGAGGTCAGTGACGATGGTCCGGGCATTCCGCCCGACGTCATGGGCAA<br>GATCTTCGACCCGTTCTTCACCACCAAGCCGGTGGGCGAGGGTACGGGCCTAGGCCTAGCCACGG<br>TCTATGGCATCGTTAAGCAGAGCGACGGCTGGATTCACGTCCACAGCCGTCCGAACGAAGGCGCG<br>GCCTTCCGCATCTTCCTGCCGGTCTATGAAGCGCCCGCCGGCGCGGTCGCCGTCCAGGCCGTCGC<br>CGAGCCCGCCAAGCCGCGCGCCGCTCGCGACCTGTCGGGCGCCGGCCGCATCCTGTTCGTCGAG<br>GACGAGGACGCCGTGCGCAGCGTCGCCGCCCGCCTGCTGCGCGCCCGTGGCTACGAGGTGCTTG<br>AGGCGGCCGACGGCGAAGAGGCCCTGATCATCGCCGAGGAAAACGCCGGCACGATCGACCTCTT<br>GATCAGCGACGTGATCATGCCCGGCATCGACGGCCCGACCTTGCTGAAGAAGGCGCGTGGCTATC<br>TGGGGACCGCGCCGGTGATGTTCATCTCCGGCTATGCCGAGGCCGAGTTCAGCGACCTCTTGGAA<br>GGCGAGACGGGCGTGACCTTCCTCCCCAAGCCGATCGACATCAAGACCCTGGCCGAGCGCGTCA<br>AGCAGCAGCTGCAAGCAGCATAGtctgtgctattctaacgaatcttgattcgTCACGGTTCAGATCCACATAG<br>GCGCGGGCGAAGCGGGCGCGCACCACGGCGGCCCCCAGGGCGGGCGCGCCGCCGATGATGCG<br>GTCGACAAACGCGTCCTCGGACGCGCGCAGGGTCTCGAGCGGCAGGCGGACCGCGGCGACCAT<br>GTCCTCCGGATAGAGGTCGCCCGAGTGCGGCGAGGCGAACACCAAGGCGGTCGGCGGCGGCGC<br>CCCGGCCGGGACCGCGCGCAGCACCTCGAACGCTGGCCCGCCAAAGGTCTCCAGCGGCGGGGA<br>CGAAAGCTCTTCGCTCGAAAGGGGCCGCCACGCGCTCATGCCGCATCATCGAAGCGTCGCGGCGC<br>GGGGTCAAATCTCAATCGCCGCTGACGAAACATCCCCAGCCGCGACGTTTAGGTTCATCCCCGATT<br>TACGGACGGGGCGATAAGGTGGATCCTCTATCGACGATCTTAATCGGACACGTGACCCCatgGCCC<br>GCATCCTCCTCGCCGAAGACGATGATTCCCTGCGCGGCTTCCTGGCCCGCGCGCTGGAACGCGCC<br>GGCTTCGAAGTCCAGGCCTGCGCCGACGGCGAAGAGGCCGTCCAGCACCTGGACCATCCCTGGG<br>ACCTGCTGCTGACCGCCATCGTCATGCCCGGCATGGACGGCATCGAGGTGGCCCGCCAGGCCGCC<br>GCCCGCGACCCGTCCCTGCGCATCATGTTCATCACCGGCTTCGCCGCCGTGGCCCTCTCGGCCCAG<br>GACCGCGCGCCCGCCGGCGCCAAGGTGCTGTCCAAGCCCGTGCACCTGCGCGACCTCGTCGCCG<br>AGGTCGAAAAGATGATGGCGGCCtgagaattcctgc

Supplementary Table 3

	Diffusion Coefficients								PopZ Microdomain Size	
	Top Cell				Bottom Cell				Top Cell	Bottom Cell
File #	Y_cyto	R_cyto	Y_pole	R_pole	Y_cyto	R_cyto	Y_pole	R_pole		
1	20	20	0.5	0.5	20	20	0.5	0.5	0.50%	0.125%
2	20	20	0.5	0.5	20	20	0.5	0.5	0.50%	2.000%
3	20	20	0.5	0.5	20	20	0.5	0.5	0.50%	0.125%
4	20	20	0.5	0.5	20	20	0.5	0.5	0.50%	2.000%
5	20	20	20	20	20	20	5	5	0.50%	0.50%
6	20	20	20	20	20	20	2	2	0.50%	0.50%
7	20	20	0.5	0.5	20	20	0.125	0.125	0.50%	0.50%
8	20	20	0.5	0.5	20	20	0.05	0.05	0.50%	0.50%
9	20	20	0.05	0.05	20	20	0.005	0.005	0.50%	0.50%
10	20	20	0.0005	0.0005	20	20	0.00005	0.00005	0.50%	0.50%
11	20	20	0.000005	0.000005	20	20	0	0	0.50%	0.50%
12	20	20	0.000005	0.000005	20	20	5E-07	5E-07	0.50%	0.50%
13	20	20	5E-08	5E-08	20	20	5E-09	5E-09	0.50%	0.50%
15	20	20	0.5	0.5	20	20	0.5	0.5	0.50%	4.000%
16	20	20	0.5	0.5	20	20	0.5	0.5	15.00%	40.000%
17	0.5	0.5	0.5	0.5	0.5	0.5	20	20	100.00%	60.000%
18	20	20	0.5	0.5	20	20	0.5	0.5	0.50%	0.000%
19	20	20	2	2	20	20	0.125	0.125	0.50%	0.50%

Particle diffusion and PopZ microdomain size parameters for Smoldyn files. Each file includes simulations for two different cells, labeled top and bottom. Y and R denote yellow and red colored particles, respectively, in bulk cytoplasm (cyto) and in PopZ microdomains (pole). PopZ microdomain size is expressed as a percentage of total cell volume.

Supplementary Table 4

File Name	Top Cell	(description)	Bottom Cell	(description)
9E	Δ*popZ*	All Difcs at pole = Difc in cytoplasm	normal	Models wildtype cell No Sub1-PopZ interaction
9I	Δ*popZ*	All Difcs at pole = Difc in cytoplasm	normal	Models wildtype cell + Sub1-PopZ interaction
9J	$RcdA^{mut}$	RcdA Difc at pole = RcdA Difc in cytoplasm	$CpdR^{mut}$	CpdR Difc at pole = CpdR Difc in cytoplasm
9K	Δ*cpdR*	Zero CpdR particles	Δ*rcdA*	Zero RcdA particles
9L	2% pole volume	Pole occupies 2% of total cell volume	1/160 Difc	All Difcs at pole = 1/160 Difc in cytoplasm

Descriptions of Smoldyn files associated with Fig. 6c and Supplementary Fig. 8c. Each file includes simulations for two different cells, labeled top and bottom. 9J, 9K, and 9L variants are changed relative to the Normal cell in File 9I, which is described in the right-hand panel in Fig. 6c. The parameters used for the Normal cell are described in Methods.

Supplementary References:

1. Evinger, M. & Agabian, N. Envelope-associated nucleoid from Caulobacter crescentus stalked and swarmer cells. *J Bacteriol* 132, 294–301 (1977).

2. Bowman, G. R. *et al.* A Polymeric Protein Anchors the Chromosomal Origin/ParB Complex at a Bacterial Cell Pole. *Cell* 134, 945–955 (2008).

3. Bowman, G. R. *et al.* Oligomerization and higher-order assembly contribute to sub-cellular localization of a bacterial scaffold. *Molecular Microbiology* 90, 776–795 (2013).

4. Ryan, K. R., Judd, E. M. & Shapiro, L. The CtrA Response Regulator Essential for Caulobacter crescentus Cell-cycle Progression Requires a Bipartite Degradation Signal for Temporally Controlled Proteolysis. *Journal of Molecular Biology* 324, 443–455 (2002).

5. Iniesta, A. A., McGrath, P. T., Reisenauer, A., McAdams, H. H. & Shapiro, L. A phospho-signaling pathway controls the localization and activity of a protease complex critical for bacterial cell cycle progression. *Proc Natl Acad Sci U S A* 103, (2006).

6. Perez, A. M. Assembly of a Sub-Cellular Protein Complex Generates Asymmetry in the Bacterium Caulobacter crescentus. (Stanford University, 2015).

7. Holmes, J. A. *et al.* Caulobacter PopZ forms an intrinsically disordered hub in organizing bacterial cell poles. *Proc Natl Acad Sci U S A* 113, 12490–12495 (2016).

8. McGrath, P. T., Iniesta, A. A., Ryan, K. R., Shapiro, L. & McAdams, H. H. A Dynamically Localized Protease Complex and a Polar Specificity Factor Control a Cell Cycle Master Regulator. *Cell* 124, 535–547 (2006).
