# Supplementary material for: Phospho-signaling couples polar asymmetry and proteolysis within a membraneless microdomain in Caulobacter crescentus
Source: Nat Commun. 2024 Oct 28;15:9282. doi: 10.1038/s41467-024-53395-y (PMC11519897; doi:10.1038/s41467-024-53395-y)
Supplement: Supplementary file 2 — Description of Additional Supplementary Information [file 41467_2024_53395_MOESM2_ESM.docx]

**Description of Additional Supplementary Files**

File Name: Supplementary Data 1

Description: Smoldyn files

File Name: Supplementary Movie 1

Description: Time-lapse video of FRAP

File Name: Supplementary Movie 2

Description: Time-lapse video of model #18.

File Name: Supplementary Movie 3

Description: Time-lapse video of model #19.

File Name: Supplementary Movie 4

Description: Time-lapse video of model #9I.
