# Supplementary material for: Phospho-signaling couples polar asymmetry and proteolysis within a membraneless microdomain in Caulobacter crescentus
Source: Nat Commun. 2024 Oct 28;15:9282. doi: 10.1038/s41467-024-53395-y (PMC11519897; doi:10.1038/s41467-024-53395-y)
Supplement: Supplementary file 8 — Reporting Summary [file 41467_2024_53395_MOESM8_ESM.pdf]

nature portfolio

Corresponding author(s): Grant R Bowman

Last updated by author(s): Oct 7, 2024

Reporting Summary

Nature Portfolio wishes to improve the reproducibility of the work that we publish. This form provides structure for consistency and transparency in reporting. For further information on Nature Portfolio policies, see our Editorial Policies and the Editorial Policy Checklist.

Statistics

For all statistical analyses, confirm that the following items are present in the figure legend, table legend, main text, or Methods section.

n/a	Confirmed	
☐	☒	The exact sample size (*n*) for each experimental group/condition, given as a discrete number and unit of measurement
☐	☒	A statement on whether measurements were taken from distinct samples or whether the same sample was measured repeatedly
☐	☒	The statistical test(s) used AND whether they are one- or two-sided<br>*Only common tests should be described solely by name; describe more complex techniques in the Methods section.*
☒	☐	A description of all covariates tested
☐	☒	A description of any assumptions or corrections, such as tests of normality and adjustment for multiple comparisons
☐	☒	A full description of the statistical parameters including central tendency (e.g. means) or other basic estimates (e.g. regression coefficient) AND variation (e.g. standard deviation) or associated estimates of uncertainty (e.g. confidence intervals)
☐	☒	For null hypothesis testing, the test statistic (e.g. *F*, *t*, *r*) with confidence intervals, effect sizes, degrees of freedom and *P* value noted<br>*Give P values as exact values whenever suitable.*
☒	☐	For Bayesian analysis, information on the choice of priors and Markov chain Monte Carlo settings
☒	☐	For hierarchical and complex designs, identification of the appropriate level for tests and full reporting of outcomes
☒	☐	Estimates of effect sizes (e.g. Cohen's *d*, Pearson's *r*), indicating how they were calculated

Our web collection on statistics for biologists contains articles on many of the points above.

Software and code

Policy information about availability of computer code

Data collection	Zen 2 pro, NMRPipe software
Data analysis	Smoldyn-2.71, Fiji, MicrobeJ, POKY software suite

For manuscripts utilizing custom algorithms or software that are central to the research but not yet described in published literature, software must be made available to editors and reviewers. We strongly encourage code deposition in a community repository (e.g. GitHub). See the Nature Portfolio guidelines for submitting code & software for further information.

Data

Policy information about availability of data

All manuscripts must include a data availability statement. This statement should provide the following information, where applicable:

- Accession codes, unique identifiers, or web links for publicly available datasets
- A description of any restrictions on data availability
- For clinical datasets or third party data, please ensure that the statement adheres to our policy

All data supporting the findings of this study are available within the paper and its Supplementary Information.

Research involving human participants, their data, or biological material

Policy information about studies with human participants or human data. See also policy information about sex, gender (identity/presentation), and sexual orientation and race, ethnicity and racism.

Reporting on sex and gender	Not applicable
Reporting on race, ethnicity, or other socially relevant groupings	Not applicable
Population characteristics	Not applicable
Recruitment	Not applicable
Ethics oversight	Not applicable

Note that full information on the approval of the study protocol must also be provided in the manuscript.

Field-specific reporting

Please select the one below that is the best fit for your research. If you are not sure, read the appropriate sections before making your selection.

[x] Life sciences [ ] Behavioural & social sciences [ ] Ecological, evolutionary & environmental sciences

For a reference copy of the document with all sections, see nature.com/documents/nr-reporting-summary-flat.pdf

Life sciences study design

All studies must disclose on these points even when the disclosure is negative.

Sample size

Fig. 1b, n>300/time point for the bar chart.
Fig. 1c, Samples from experiment shown in 1b.
Fig. 1e, Representative images.
Fig. 1f, Samples from experiment shown in 1e,f.
Fig. 1g, Representative image.
Fig. 1h, n>300/types of cell.
Fig. 2b, Representative images; n=60 per cell types for the graphs.
Fig. 2c, Samples from experiment shown in 2b.
Fig. 2d, Representative images; n=10 per cell types for the graphs.
Fig. 3a, Not applicable
Fig. 3b, Representative images.
Fig. 3c, Samples from experiment shown in 2d.
Fig. 3d, n>20 per types of condensates.
Fig. 4c, Representative images.
Fig. 4d, n>300/types of cell.
Fig. 5a, Representative images.
Fig. 5b, Representative images; n=20 per cell types for the graphs.
Fig. 5c, Representative gel images; n=3 for the graphs.
Fig. 6a-c, Not applicable.
Supplementary Fig. 1a, n=2
Supplementary Fig. 1b, n=3
Supplementary Fig. 1c, n=3
Supplementary Fig. 2a, n=3
Supplementary Fig. 2b, Representative images; n=60 per cell types for the graphs.
Supplementary Fig. 2c, Representative images.
Supplementary Fig. 2d, Representative images; n=10 per cell types for the graphs.
Supplementary Fig. 3a-b, Not applicable.
Supplementary Fig. 4 a-c, Representative images.
Supplementary Fig. 5a, Representative images, n=20 per cell types for the graph.
Supplementary Fig. 5b, Representative images, n=20 per cell types for the graph.
Supplementary Fig. 6a, Representative gel images, n=3 for the graphs.
Supplementary Fig. 7a, n=3.
Supplementary Fig. 8a-c, Not applicable.

Data exclusions

4 c, Last two time points were not shown in the graphs as the signals were no higher than the background.

Replication

Fig. 1b, 5-10 images from a single representative experiment which was repeated two times and produced similar results;
Fig. 1c, Experiment was repeated two times and produced similar results.

Fig. 1e, Experiment was repeated three times and produced similar results.
Fig. 1f, Experiment was repeated three times and produced similar results.
Fig. 1g, Experiment was repeated three times and produced similar results.
Fig. 1h, Experiment was repeated three times and produced similar results.
Fig. 2b, Experiment was repeated three times and produced similar results.
Fig. 2c, Experiment was repeated three times and produced similar results.
Fig. 3b, Experiment was repeated three times and produced similar results.
Fig. 3c, Experiment was repeated three times and produced similar results.
Fig. 4c-d, Experiment was repeated three times and produced similar results.
Fig. 5a, Experiment was repeated three times and produced similar results.
Fig. 5b, Experiment was repeated three times and produced similar results.
Fig. 5c, Experiment was repeated three times and produced similar results.
Fig. 6a-c, Representative data from individual runs. In this model, we observed no significant variation between runs initiated with same parameters.
Supplementary Fig. 1a-c, Experiment was repeated three times and produced similar results.
Supplementary Fig. 2a-b, Experiment was repeated three times and produced similar results.
Supplementary Fig. 2d, Experiment was repeated three times and produced similar results.
Supplementary Fig. 4a-c, Experiment was repeated three times and produced similar results.
Supplementary Fig. 5a-b, Experiment was repeated three times and produced similar results.
Supplementary Fig. 8a-b, Representative data from individual runs. In this model, we observed no significant variation between runs initiated with same parameters.

Randomization: Not applicable

Blinding: Not applicable

Reporting for specific materials, systems and methods

We require information from authors about some types of materials, experimental systems and methods used in many studies. Here, indicate whether each material, system or method listed is relevant to your study. If you are not sure if a list item applies to your research, read the appropriate section before selecting a response.

Materials & experimental systems

n/a	Involved in the study
☐	☒ Antibodies
☒	☐ Eukaryotic cell lines
☒	☐ Palaeontology and archaeology
☒	☐ Animals and other organisms
☒	☐ Clinical data
☒	☐ Dual use research of concern
☒	☐ Plants

Methods

n/a	Involved in the study
☒	☐ ChIP-seq
☒	☐ Flow cytometry
☒	☐ MRI-based neuroimaging

Antibodies

Antibodies used: HA Epitope Tag, Invitrogen (2-2.2.140), Ref 26183, lot YB362804; GFP antibody, Gene tex GTX113617, Lot 42179. HRP conjugated secondary antibody Jackson ImmunoResearch.

Validation: Not applicable

Plants

Seed stocks: Not applicable

Novel plant genotypes: Not applicable

Authentication: Not applicable
